# Reversing A*β* Fibrillation and Inhibiting A*β* Primary Neuronal Cell Toxicity Using Amphiphilic Polyphenylene Dendrons

**DOI:** 10.1002/adhm.202101854

**Published:** 2021-11-19

**Authors:** Siyuan Xiang, Jessica Wagner, Thorsten Lückerath, Klaus Müllen, David Y. W. Ng, Jana Hedrich, Tanja Weil

**Affiliations:** ^1^ Max Planck Institute for Polymer Research Ackermannweg 10 Mainz 55128 Germany

**Keywords:** Alzheimer's disease, A*β*, blood–brain barrier delivery, dendrimers, protein–polymer hybrid materials

## Abstract

Uncontrolled amyloid‐beta (A*β*) fibrillation leads to the deposition of neurotoxic amyloid plaques and is associated with Alzheimer's disease. Inhibiting A*β* monomer fibrillation and dissociation of the formed fibers is regarded as a promising therapeutic strategy. Here, amphiphilic polyphenylene dendrons (APDs) are demonstrated to interrupt A*β* assembly and reduce A*β*–cell interactions. Containing alternating negatively charged sulfonic acid and hydrophobic n‐propyl peripheral groups, APDs bind to the secondary structure of the A*β* aggregates, inhibiting fibrillation and disassemble the already formed A*β* fibrils. APDs reveal vesicular cellular uptake in endosomes as well as cell compatibility for endothelial and neuronal cells, and significantly reduce A*β*‐induced neuron cytotoxicity in vitro. Moreover, they are transported into the brain and successfully cross the blood–brain barrier after systemic application in mice, indicating their high potential to inhibit A*β* fibrillation in vivo, which can be beneficial for developing therapeutic strategy for Alzheimer's disease.

## Graphical abstract

Amyloid‐beta (A*β*) assembly plays essential roles in Alzheimer's plaque formation. Amphiphilic polyphenylene dendrons (APDs) with their bioconjugation (SA‐D), which inhibit A*β* fibrillation, disassemble the already formed fibrils, and significantly reduce the A*β*‐induced cytotoxicity, are presented here. APDs and SA‐D reveal specific neurons uptake and cross the blood–brain barrier in vivo, providing new opportunities as therapeutic candidates for Alzheimer's disease.

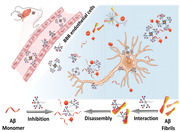

## 1 Introduction

Abnormal folding and aggregation of peptides or proteins contributes to the pathology of a range of neurodegenerative diseases, like Alzheimer's, Parkinson's, Huntington disease, and amyotrophic lateral sclerosis. The disease impact for patients, families, and the economy is dramatic, yet, efficient drugs to reverse or stop the pathology of amyloid diseases are still lacking. Therefore, the investigation of amyloid pathology and new therapeutic interventions is highly relevant.^[^ ^1^ ^]^ Following the amyloid hypothesis in Alzheimer's,^[^ ^2^ ^]^ the appearance of amyloid‐beta (A*β*) peptide with high concentrations in brain parenchymal is an early toxic event in the pathogenesis of AD. The amyloid accumulation is followed by neuroinflammation, tau accumulation, metabolism and synaptic dysfunction, neuronal death, and cognitive decline.^[^ ^2^, ^3^ ^]^ Therefore, drugs inhibiting the early step of oligomer formation could affect AD progression, before the cascade of multiple pathological effects occurs.^[^ ^3^ ^]^ Another challenge for neuropharmarceutical approaches is the development of drugs with adequate BBB permeability, which is essential for the treatment of chronic diseases.^[^ ^4^ ^]^ Furthermore, it is crucial for the drugs to perform a noninvasive passage through the blood–brain barrier (BBB), in order to reduce side effects. Consequently, a promising strategy has to combine antiamyloid properties as well as efficient BBB transport.

Preclinical antiamyloid strategies have been investigated, as A*β* fibrils occur even before the first appearance of clinical symptoms where neurons are irreversibly damaged. However, the amount of amyloid plaques does not correlate to the disease severity, suggesting that the oligomeric and protofibrillary structures trigger a neurotoxic cascade.^[^ ^2^, ^3^ ^]^ Therefore, recent discussions indicate that antiamyloid strategies targeting toxic oligomeric A*β* aggregation would be most beneficial in the early stages of AD.^[^ ^3^ ^]^ Several therapeutic approaches reached preclinical development stages, which focus on the reduction of amyloid oligomer levels or the breaking of formed *β*‐sheet fibrils.^[^ ^3^ ^]^ Current strategies also included antibodies for the neutralization of oligomeric species, overexpression of A*β*‐degrading enzymes, catalytic antibodies, small molecule inhibitors, and *β*‐sheet blockers.^[^ ^3^, ^5^ ^]^ In addition, several concepts on AD treatment based on dendrimers functioning as *β*‐sheet blockers have been developed.^[^ ^6^ ^]^

Dendrimers are macromolecules consisting of a central core, a shell, and periphery. Each segment starting from the core is called a dendron and each layer of the dendrimer is termed generation. With increasing generation, the size and branching degree of the dendrimer are enlarged, leading to more functional groups on its periphery.^[^ ^7^ ^]^ For example, dendrimers consisting of a poly(propylene)imine, poly(amidoamine) (PAMAM), phosphorus or poly(lysine) scaffold, and positively charged terminal groups interact with A*β* fibrils, which affect aggregation and toxicity of A*β*.^[^ ^8^ ^]^ However, cationic dendrimers have several limitations, as they are quickly eliminated by both the kidney and liver as well as inducing cytotoxicity at high concentrations/generations due to electrostatic interactions with cellular membranes resulting in nanopores and thereby membrane leakage.^[^ ^9^ ^]^ To enhance blood‐circulation times and to lower the cellular toxicity, anionic and neutral dendrimers are preferable.^[^ ^10^ ^]^

**Figure 1:**
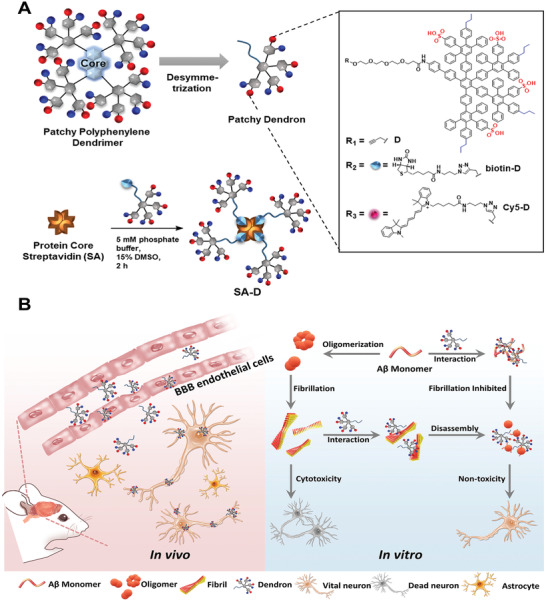
Schematic overview of the study. A) Amphiphilic PPDs and the design of the corresponding APDs: D, biotin‐D, and Cy5‐D consisting of only one dendritic branch of the entire PPD. The propargyl group at the focal point of D allowed functionalization with d‐biotin and the dye Cy5 to afford biotin‐D (2) and Cy5‐D (3).^[^ ^13^ ^]^ Four equivalents of biotin‐D (2) are assembled to SA. B) In vivo uptake of amphiphilic dendron (D) was found mainly for endothelial cells and neurons. In vitro antiamyloidogenic activity of D was studied by incubating with A*β* (1‐42) peptide. Inhibition of fiber formation as well as disassembly of formed A*β* fibrils was observed. These A*β*‐amphiphilic dendron interactions inhibited cytotoxicity of A*β* fibrils in primary murine neuronal cells.

To address these challenges, negatively charged amphiphilic (also denoted as patchy) polyphenylene dendrimers (PPDs) consisting of alternating sulfonic acid and n‐propyl groups have been designed and synthesized.^[^ ^11^ ^]^ Previously, the internalization of amphiphilic PPDs into brain endothelial cells was presented hereby showing their potential for brain delivery.^[^ ^11b^ ^]^ A second generation amphiphilic PPD showed a binding to the protein‐based capsid of adenovirus 5 (Ad5), thus forming a new dendrimer corona. The new PPD corona re‐directed the Ad5 biodistribution both in vitro and in vivo and facilitated uptake into cancer cells, which offers the potential for virus‐assisted gene therapy.^[^ ^12^ ^]^ We have shown previously that the respective APD (**Figure** **1**A) possesses the same cellular uptake and virus binding features as the full PPD. Thus, polyphenylene dendrons could be considered as minimal fragments of a globular PPD, revealing similarly high cell viability, interactions with blood serum proteins and virus binding as the full dendrimer. Moreover, dendrons have the advantage that they can be modified easily at their core providing the potential to attach dyes, proteins, or nanoparticles.^[^ ^13^ ^]^

In this study, we synthesized APDs with a lipophilic propargyl group (D) and with terminal D‐biotin (biotin‐D),^[^ ^13^ ^]^ where we assembled four biotin‐D onto the protein streptavidin to afford the dendronized protein SA‐D, as depicted in Figure 1A. Taking advantage of the tetrameric streptavidin core, we conceptualized that SA‐D would present itself as a highly attractive dendron delivery system and also a protein scaffold for further multiple functionalization in the future. We investigated the effects and potency of D and SA‐D in the inhibition of A*β* fibril formation and whether multivalency interactions play a role in the disassembly of already formed fibrils. We also studied the potential of APDs to reduce A*β*–cell interactions, inhibiting cytotoxicity in primary neuronal cells and to pass the BBB in vivo. In validating the capacity of D and SA‐D to successfully reverse A*β* fibrillation and toxicity, together with transport across the BBB in mice, we envision that D and SA‐D have a high drug like potential which could be beneficial for the treatment of A*β* aggregation in Alzheimer's disease (Figure 1B) and as therapeutics towards other protein‐misfolding‐induced disorders in the future.

## 2 Results and Discussion

### 2.1 Synthesis and Characterization of D and SA‐D

**Figure 2:**
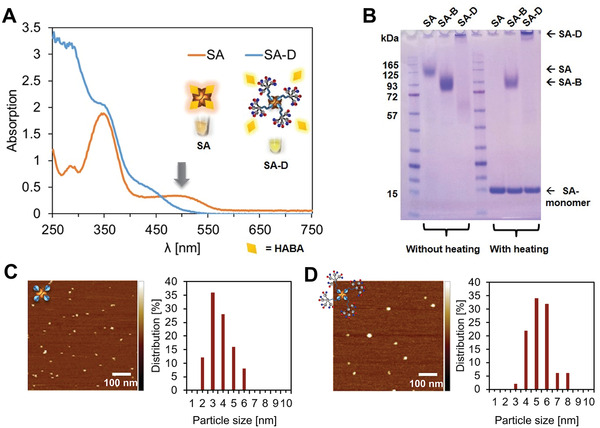
Characterization of SA‐D. A) HABA‐assay shows a distinct absorption band at 500 nm for SA, which is reduced for SA‐D, indicating that all four binding sites have been occupied by biotin‐D. B) SDS‐PAGE under heating and nonheating conditions shows the formation of SA‐D and SA‐B, respectively. C,D) AFM topographic images reveal an increase in height after formation of SA‐D (C) in comparison to SA‐B (D).

The synthesis of APD with a propargyl‐moiety (D), a d‐biotin (biotin‐D) and a Cy5 chromophore (Cy5‐D) was accomplished as reported previously and as depicted in Figure 1A.^[^ ^13^ ^]^ The chemical structures and comprehensive characterization of all dendron conjugates applied herein are shown in Figure S1 of the Supporting Information. Four biotin‐Ds were then assembled on the protein streptavidin (SA) (Figure 1A). SA is a homotetrameric protein with four biotin‐binding sites, and the binding affinity of d‐biotin to streptavidin is among the strongest interactions (*K*_d_ ≈ 10^−14^ m) in nature.^[^ ^14^ ^]^ The assembly of four biotin‐D molecules on streptavidin (Figure 1A) affords SA‐D with an SA protein core and an amphiphilic dendron shell. In this way, the impact of multiple dendrons on bioactivity could be studied in a straightforward fashion. Moreover, SA is often used as a platform in drug delivery as it allows easy preparation of nanocarriers with drugs and targeting or imaging molecules by simple self‐assembly in solution.^[^ ^15^ ^]^ Therefore, SA‐D could be easily equipped with other substituents such as dyes for labeling without affecting the efficacy of the attached dendrons. SA‐D was prepared by the addition of biotin‐D in DMSO to a solution of SA (or Cy5‐labeled SA (Cy5‐SA‐D) for imaging experiments) (Figures S2–S4, Supporting Information) in phosphate buffer at pH 7.4. After incubation of the SA‐D mixture for 2 h, SA‐D was purified by size exclusion chromatography (SEC) to remove excessive amounts of biotin‐D. Cy5‐SA‐D (Figures S2–S4, Supporting Information) was prepared and purified following the same procedure. SA complexed with free d‐biotin (SA‐B) served as a reference (Figure S5, Supporting Information). The occupation of d‐biotin binding sites of SA by biotin‐D was verified by the addition of 2‐[(4‐hydroxyphenyl)‐diazenyl]benzoic acid (HABA). HABA binds to free d‐biotin binding sites resulting in an absorption band at 500 nm (orange line).^[^ ^16^ ^]^ When adding HABA to SA‐D, this absorption band was absent, as also observed for SA‐B (green line) (**Figure** **2**A; Figure S6, Supporting Information). Moreover, an increase in absorption in the UV region (≈250–350 nm) alludes to the presence of the aromatic polyphenylene dendrons (Figure 2A). These findings indicate the occupation of biotin‐binding sites by biotin‐D. Besides, sodium dodecyl sulfate polyacrylamide gel electrophoresis (SDS‐PAGE) analysis of SA‐D without and with heating and addition of the denaturation agent 1,4‐dithio‐d‐threitol confirms the binding of biotin‐D to SA (Figure 2B). The tetrameric SA‐D complex mainly remained in the loading wells without the employment of heat, which is due to aggregation of the SA‐D conjugate in the MES running buffer. The SA monomer, SA‐B, and SA‐D tetramers are only separated after heating, which denature **SA** so that it disassembles into monomers. Compared to **SA**, the disassembly of SA‐B and SA‐D was dramatically reduced. Tetramers coexisted with the monomers in the gel were observed, since biotin‐binding increases the thermal stability and resistance of SA against denaturing agents.^[^ ^17^ ^]^ The successful attachment of the biotin‐D to SA was also verified by agarose gel electrophoresis (Figure S7, Supporting Information). In contrast to SA and SA‐B, the SA‐D conjugate did not show any migration in the gel during agarose gel electrophoresis and SDS‐PAGE. In addition, in atomic force microscopy (AFM) and dynamic light scattering (DLS) experiments (Figure 2D; Table S1, Supporting Information), SA‐D revealed larger sizes compared to the reference SA‐B (Figure 2C). In 25 × 10^−3^ m phosphate buffer at pH 7.4, a hydrodynamic radius (*R*_h_) of 3.3 nm for SA‐B and 25.1 nm for SA‐D (Table S1, Supporting Information) were measured. The ionic strength of the solution also had a significant effect on the *R*_h_ of SA‐D. The application of 50 × 10^−3^ m phosphate buffer (PB) at pH 7.4 further decreased the *R*_h_ of SA‐D to 23.8 nm, whereas ultrapure water or 100 × 10^−3^ m PB resulted in larger *R*_h_ values of 37.3 and 32.8 nm, respectively. The height topographic images obtained from AFM showed an expected size increase from ≈3 nm for SA‐B to ≈5–6 nm for SA‐D (Figure 2C,D). Typical polyphenylene dendrimer–protein conjugates always required the addition of detergents to solubilize the hydrophobic polyphenylene scaffold. ^[^ ^18^ ^]^ By contrast, SA‐D and Cy5‐SA‐D were dissolved in aqueous solution after SEC without any auxiliaries such as detergents with concentrations up to 17 × 10^−6^ and 30 × 10^−6^ m, respectively.

### 2.2 Inhibition of A*β* Fibrillation

Protein aggregates such as *β*‐amyloid fibrils are formed by noncovalent interactions such as hydrogen‐bonds as well as van der Waals interactions. In the next step, we investigated whether the APDs could interact with A*β* (1‐42) to inhibit the fibrillation process. Thioflavin T (ThT) was selected as a fluorescent probe to monitor the A*β* fibrillation process with and without the amphiphilic dendrons D and SA‐D. ThT binds specifically to the *β*‐sheets of fibrils, which is observed by a significant increase in fluorescence intensity due to the rotational immobilization of the central C—C bond in ThT connecting the benzothiazole and aniline rings^[^ ^19^ ^]^ during binding to the side chain channels along the axis of the fibrils.^[^ ^20^ ^]^ The kinetics of the fibrillation process of A*β* (1‐42) was studied, as shown in Figure S8A of the Supporting Information. A*β* monomers of different concentrations were incubated with ThT at 37 ˚C for 16 h. After 1 h lag phase, an exponential increase of the fluorescence intensity started, indicating that for the supramolecular polymerization of A*β*, the nucleation process ended after 1 h. Nuclei (A*β* oligomers) as well as monomers started to form the *β*‐sheet containing fibrils in the elongation phase (exponential phase of the sigmoid curve).^[^ ^21^ ^]^ After 45 h, the fluorescence intensity reached a plateau, which was maintained for 16 h, indicating that the fibrillation process was complete after 5 h (Figure S8A, Supporting Information). The morphologies of the A*β* in different stages were monitored by transmission electron microscopy (TEM) imaging. The peptide monomers could not be imaged in TEM (Figure S8B, Supporting Information), while the A*β* oligomers showed particle‐like and amorphous structures (Figure S8C, Supporting Information). After 16 h, long fibrils were observed (Figure S8D, Supporting Information). These data indicated the time scale for the fibrillation process and confirmed the formation of A*β* fibrils.

The impact of D and SA‐D at different ratios on A*β* fibrillation is presented in Figure S9A,B of the Supporting Information. After the application of an equimolar ratio of D and A*β* (D:A*β* = 1:1), a decrease of the slope in the exponential phase was observed, indicating a slowdown of the elongation process and less *β*‐sheet formation. Further increasing the amount of A*β* (D:A*β* = 1:5 and 1:8) still inhibited the elongation phase and the final amount of A*β* fibrils. For the 1:8 ratio, the final plateau showed a decrease to 491.8% compared to 739.8% for A*β* alone (dark magenta line and gray line, Figure S9A, Supporting Information), indicating that the fibrillation process was effectively inhibited and delayed even with stoichiometric excess of A*β*. Since one SA‐D molecule binds four dendrons, fibril formation is completely inhibited at the equimolar ratio of SA‐D to A*β* (red line, Figure S9B, Supporting Information). With the higher molar ratio of A*β* (SA‐D:A*β* = 1:5), the fibril elongation phase was still inhibited, and for SA‐D:A*β* at 1:8, a decrease in the final fibril concentration to 490.6% was observed (dark magenta line, 739.8% for A*β* alone, Figure S9B, Supporting Information). TEM imaging results were also consistent with the kinetics experiments. At equimolar ratios, fibrillation was inhibited, whereas at higher A*β* concentrations, fibrils could be observed (Figure S9, Supporting Information).

**Figure 3:**
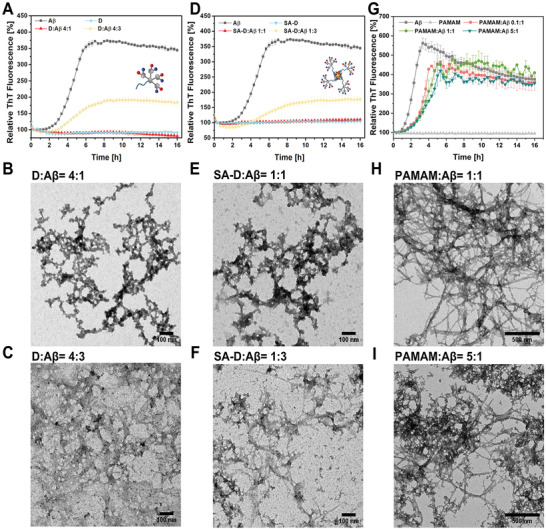
A–I) Inhibiting A*β* fibrillation with D, SA‐D, and PAMAM dendrimer G3. ThT kinetics of A*β* with D (A), D) SA‐D (D), and G) PAMAM G3 (G) at different molar ratios (in all ratios, the A*β* concentration is 5 × 10^−6^ m), and the corresponding TEM images of the mixture. The scale bar for (B), (C), (E), and (F) is 100 nm, for (H) and (I) is 500 nm. (A,D,G) Data are presented as mean with standard deviation (SD), *n* = 3.

Then, we kept the same molar ratios of D and SA‐D to A*β*, in terms of dendron content. Attributed to the fact that four dendrons are attached to SA, four molar equivalents of D compared to SA‐D were applied as described in the material and methods section in greater detail. After 2 h, the fluorescence intensity of A*β* alone started to increase and reached a plateau at ≈5–6 h. During incubation of D and A*β* in the molar ratio 4:1 (D:A*β*) there was no pronounced increase in fluorescence intensity even after an incubation of 16 h (**Figure** **3**A, red line). Using equimolar concentrations of SA‐D and A*β* (SA‐D:A*β* = 1:1), also no fluorescence intensity increase was observed during the 16 h incubation time (Figure 3D, red line). These data suggested that fibrillation of A*β* was inhibited by both D and SA‐D at dendron to A*β* ratio of 4:1 and SA‐D to A*β* ratio of 1:1. Increasing the amount of A*β* to 4:3 (D:A*β*) or 1:3 (SA‐D:A*β*), the slope of the elongation phase was still decreased, compared to A*β* alone (349.3%), accordingly, the formed plateau was reduced to 181.7% and 176%, respectively. Both D and SA‐D revealed a similar inhibition effect on A*β* fibrillation. TEM images of the mixture also confirmed the kinetics results. After the experiments, no ordered fibrils but some amorphous structures were observed for D:A*β* (4:1, Figure 3B) and SA‐D:A*β* (1:1, Figure 3E) and few fibrils for D:A*β* (4:3, Figure 3C) and SA‐D:A*β* (1:3, Figure 3F), indicating that D and SA‐D effectively inhibited or retarded A*β* fibrillation at equimolar concentrations.

As a control experiment, SA‐B was also incubated with A*β*, and the fibrillation kinetics was assessed (Figure S10, Supporting Information). After 16 h, SA‐B:A*β* solutions revealed similar fibril formation kinetics as for A*β* alone with an even higher relative intensity of the plateau. The increase of fluorescence intensity indicates that SA‐B alone did not inhibit A*β* fibrillation, but it may rather contribute to the fibrillation process. Amyloid fibrils were observed in TEM images of both SA‐B:A*β* ratios of 1:1 and 1:3, respectively. We proved that SA‐B has no inhibiting effect on A*β* fibrillation, and the inhibiting behavior of SA‐D was caused by the amphiphilic dendrons. We also studied and compared the effect of A*β* fibrillation with polycationic PAMAM dendrimers of the third generation (G3), which were reported to modulate the amyloid formation.^[^ ^22^ ^]^ As shown in the fibrillation kinetics (Figure 3G), PAMAM incubated with A*β* affected the nucleation and elongation phase of A*β* fibrillation but there was no significant effect on the final plateau. Even at a high PAMAM to A*β* ratio of 5:1, no inhibition of fibrillation process was observed, and A*β* fibrils were seen in TEM (Figure 3H,I). In a previous study, Klajnert et al. reported an inhibition behavior of PAMAM dendrimers (G3) for A*β* peptide 1–28 at pH 5.5, and they described a reduction of the elongation rate and the final fibrillation for PAMAM.^[^ ^22^ ^]^ However, for the full‐length A*β* peptide (1‐42) at a physiological pH of 7, we could also observe the effect on the slope of the elongation phase, but no further reduction of the fibrils. These differences could be explained by different A*β* peptides and pH conditions used. In our study, the herein presented amphiphilic polyphenylene dendrons D and SA‐D significantly inhibited the fibrillation of full‐length A*β* at physiological pH (Figure 3A,D) compared to PAMAM G3 (Figure 3G).

### 2.3 Disassembly of Already Formed A*β* Fibrils

**Figure 4:**
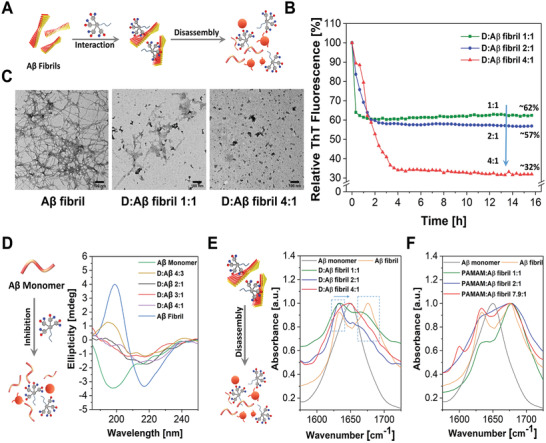
Disassembly performance of D and SA‐D on A*β* fibril. A,D) Schematic illustration of D and SA‐D disassembling the already formed A*β* fibril. B) ThT kinetics of dendron interacting with A*β* fibril at different molar ratios (in all ratios, the A*β* fibril concentration is 20 × 10^−6^ m). C) TEM images of the preformed A*β* fibrils; A*β* fibril mixed with D and SA‐D at different molar ratios. (D) Schematic illustration of A*β* fibrillation inhibition (left) and circular dichroism spectra of A*β* monomer, A*β* fibril, and D incubated with monomer at different molar ratios. E) Schematic illustration of A*β* fibril disassembly (left) and ATR FT‐IR spectra of D with A*β* fibrils at different ratios. F) ATR FT‐IR spectra of PAMAM dendrimer G3 with A*β* fibrils at different molar ratios. The scale bar for TEM images is 100 nm.

In most cases, when AD is diagnosed, amyloid plaques already exist and generate toxicity to neurons. Therefore, eliminating the already formed plaques is a critical property for therapeutic candidates. However, few molecules have been reported to disrupt the preformed A*β* aggregates.^[^ ^23^ ^]^ To investigate whether D and SA‐D could reverse fibrillation, A*β* fibrils were generated and incubated with D or SA‐D, and the results are presented in **Figure** **4** and Figure S11 (Supporting Information). ThT kinetics results clearly showed that after mixing the dendron with A*β* fibrils, the fluorescence intensity decreased over time and reached a plateau (Figure 4B). The fluorescence intensity reduced by about 38% at a ratio of D:A*β* fibril of 1:1 and decreased by 43% at a higher ratio of D (D:A*β* fibril = 2:1). Further increase of the concentration of D to 4:1 (D:A*β* fibril), resulted in a fluorescence intensity drop by 68%, indicating that already formed A*β* fibrils could be disassembled by the dendron, as illustrated in Figure 4A. The samples were also characterized via TEM. After incubation of equimolar ratios of A*β* fibrils with D (D:A*β* fibril = 1:1), considerably less A*β* fibrils were detected, but few fibrillar structures remained (Figure 4C). By increasing the amount of D (D:A*β* fibril = 2:1), nearly no A*β* fibrils but some amorphous structures were observed (Figure 4C). When the amount of D was further increased, no fibril was observed in the TEM image, indicating consistent results with ThT kinetics. For the incubation of SA‐D, all A*β* fibers disappeared, and only amorphous structures remained at ratios of 1:1 and 2:1 (SA‐D:A*β* fibril, Figure S11, Supporting Information). These results clearly show that D and SA‐D not only inhibit A*β* fibrillation but are also able to disassemble the preformed A*β* fibrils (Figure 4A; Figure S11A, Supporting Information).

### 2.4 Mechanism of Dendrons Inhibiting A*β* Fibrillation and Disassembly of Already Formed Fibrils

**Figure 5:**
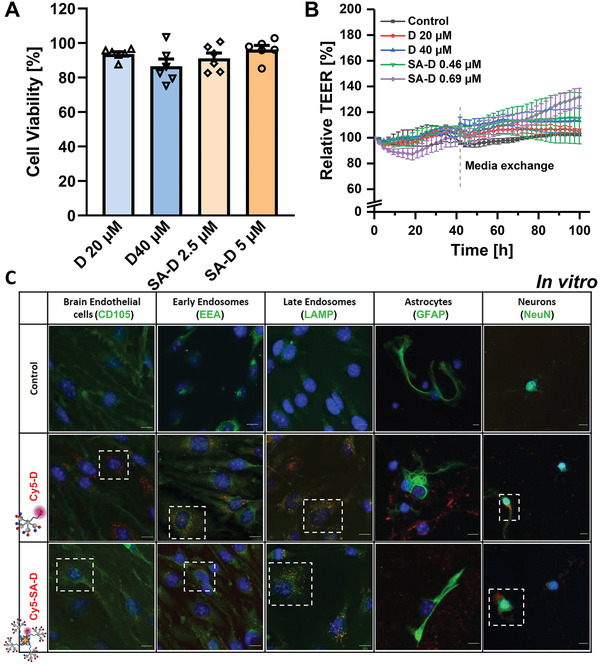
Effects of D and SA‐D toxicity, BBB integrity, and cell uptake for brain endothelial cells and primary neuronal cells. A) Cell viability of bEnd.3 cells after treatment of D and SA‐D with different molar concentration for 24 h. Data are presented as mean with SEM (standard error of the mean), *n* = 6. B) BBB integrity in vitro was investigated of D and SA‐D on bEnd.3 cell monolayer. TEER was measured by CellZscope (nanoAnalytics GmbH, Germany) and the values at *t* = 0 were set to 100% and each following measurement was expressed in relative percentage. TEER absolute values in a range between 9 and 21 Ω cm^2^, with the mean of cell layer capacitance (Ccl) of 0.91 ± 0.16 µF cm−^2^, indicating the cell confluence. Data are presented as mean with SEM, *n* = 4. C) Confocal imaging of Cy5‐D and Cy5‐SA‐D uptake in brain endothelial and neuronal cells in vitro. Cells were fixed, stained with cell‐specific antibody markers for endothelial cells (CD105, green), astrocytes (GFAP, green), early endosomes (EEA, green) or late endosomes (LAMP, green) for endothelial cells (bEnd.3), and neurons (NeuN, green). Nuclei were stained with DAPI (blue). Examples of Cy5‐D and Cy5‐SA‐D positive cells are highlighted in boxes with dashed lines. The scale is 10 µm.

Next, the inhibition mechanism of D on A*β* fibrillation was studied by circular dichroism (CD) spectroscopy. As depicted in Figure 4D, the A*β* monomers exhibited a negative peak around 198 nm, indicating the random coil structure.^[^ ^24^ ^]^ For A*β* fibrils, the negative peak disappeared and new peaks (a maximum ellipticity at around 198 nm and a minimum in the ellipticity at 216 nm) were observed, proving that the random structure of the peptide was converted to *β*‐sheets as reported previously.^[^ ^25^ ^]^ After incubating D and A*β* monomers at 4:3 (D:A*β*) ratio, a maximum ellipticity at 198 nm was still observed, but with lower intensity than A*β* fibril alone, suggesting that A*β* still partially formed the *β*‐sheets (**Figure** **5**A, yellow line). After increasing the amounts of D from 2:1 to 4:1 (D:A*β*), no *β*‐sheet bands could be observed. These results indicate that the A*β* fibrillation inhibition is most likely based on a direct interaction of D with the secondary structure of the A*β* fibrils. The observed loss of *β*‐sheets was consistent with the kinetics results (Figure 3A). At lower D:A*β* concentrations (e.g., D:A*β* 4:3), the fibrillation process of monomer was only partially inhibited, and *β*‐sheet structures were still formed as observed by the increase in ThT fluorescence intensity and the CD signals for *β*‐sheets. At high D:A*β* molar ratios (e.g., 4:1), A*β* fibrillation was fully inhibited, and neither fluorescence increase nor *β*‐sheet formation in CD was observed (Figure 4D, purple line). The disassembly process of A*β* fibrils incubated with D was then studied by ATR FT‐IR, as shown in Figure 4E. Instead of the single peak around 1648 cm^−1^ for the unordered structure of A*β* monomer (Figure 4E, gray line), two typical peaks at 1635 and 1675 cm^−1^ were observed in A*β* fibrils (Figure 4E, yellow line), indicating the formation of the secondary structure of antiparallel *β*‐sheets.^[^ ^26^ ^]^ When incubated with D at molar ratio 1:1 and 2:1, the peaks around 1675 cm^−1^ decreased, indicating the disassembly of *β*‐sheets. It is worth noting that the peaks at 1635 cm^−1^ remained. This is because the fibrils were not disassembled completely, which is consistent with the kinetics results (Figure 4B). When increasing the D:fibril to 4:1, both peaks attributed to *β*‐sheets disappeared, replaced by one peak around 1640–1647 cm^−1^, indicating that A*β* fibrils were disassembled by the dendron (Figure 4E, red line). TEM image of D:fibril at 4:1 also confirmed the result (Figure 4C). We assume that the hydrophobic patches of the polyphenylene dendrons D (phenyl rings and n‐propyl groups) interact with hydrophobic domains of A*β* (amino acids LVFFA, IIGLM). Upon binding, the repulsion between the negatively charged sulfonate groups of dendrons and the anionic peptide residues could induce the observed unfolding and defibrillation of the A*β* peptide.^[^ ^24^ ^]^ Furthermore, the aromatic amino acids of A*β* could interact with the aromatic groups of D by *π*–*π* stacking, which could inhibit fibrillation and *β*‐sheet formation of A*β*.^[^ ^24^ ^]^ In order to further elucidate the broader application of amphiphilic dendrons, we have studied the impact on another amyloid‐like peptide, Fmoc‐ISA (Figure S12A, Supporting Information), which forms *β*‐sheet rich nanofibers and induces apoptosis.^[^ ^27^ ^]^ Fmoc‐ISA is a more hydrophobic oligopeptide and corresponding monomers only dissolve in DMSO, whereas fibril formation occurs rapidly in aqueous solution compared to A*β* (1‐42) (Figure S12C, Supporting Information). We have studied the kinetics of Fmoc‐ISA monomer, Fmoc‐ISA:D mixture (1:1), and the assembled Fmoc‐ISA fibers with subsequent addition of D (1:1 ratio) using proteostat protein aggregation assay. Fmoc‐ISA forms fibrils in aqueous solution after 10–20 min, which is detected by an increase in the fluorescence intensity of proteostat (red line, Figure S12B, Supporting Information) as well as in TEM images (Figure S12C, Supporting Information). When Fmoc‐ISA monomer is incubated with D (equimolar ratio), no obvious increase of fluorescence intensity was observed (green line, Figure S12B, Supporting Information), indicating that fibrillation of Fmoc‐ISA was completely inhibited by D. TEM images also clearly show that no fibril formation was observed after 32 h in the kinetic experiments (Figure S12, Supporting Information). Moreover, already formed Fmoc‐ISA fibrils were dissociated after the addition of D into the Fmoc‐ISA solution after fibrillating for 16 h. A significant decrease of the proteostat fluorescence intensity was observed (blue line, Figure S12B, Supporting Information), suggesting the disassembly of Fmoc‐ISA fibrils. The corresponding TEM image also proved that no fibrils but only residual amorphous structures were imaged after subsequent addition of D to the Fmoc‐ISA solution (Figure S12C, Supporting Information). As a comparison, PAMAM dendrimers G3 were also applied to check the disassembly of preformed fibrils. The mixture was characterized by ATR FT‐IR (Figure 4F). After incubation of fibrils with PAMAM (G3) at different molar ratios, the peaks attributed to *β*‐sheet remained, with lots of fibrils still observed in the corresponding TEM images (Figure S13, Supporting Information), suggesting that PAMAM could not effectively disassemble the fibril even when the ratio reached 7.9:1 (PAMAM:A*β* fibril). All these data demonstrate that D and SA‐D play a leading role in inhibiting the *β*‐sheet formation, and disassemble already formed A*β* fibrils, which could make them promising candidates for the treatment of amyloid‐based disorders.

### 2.5 Biocompatibility, Effects on BBB Integrity and Target Cell Uptake

The impact of the dendrons on cell viability as well as sufficient uptake into target cells is crucial for further development. Therefore, we studied the impact of D and SA‐D on relevant endothelial cells as well. bEnd.3 cells (ATCC CRL‐2299, Manassas, VA, USA) treated with high D concentrations (20 × 10^−6^ and 40 × 10^−6^ m) resulted in 93.7 ± 1.3% and 86.6 ± 4.2% cell vitality (Figure 5A). Incubation of SA‐D (2.5 × 10^−6^ m, (binding 10 × 10^−6^ m biotin‐D), 5 × 10^−6^ m (binding 20 × 10^−6^ m biotin‐D) resulted in a cell vitality of 91.1 ± 3.1% and 96.3 ±2.4%, respectively (Figure 5A).

For brain delivery, not only the cell viability of endothelial cells is important, but also the impact on the barrier integrity. BBB integrity was tested in an in vitro model measuring transendothelial electrical resistance (TEER) with the cellZscope system (nanoAnalytics). After 24 h, the relative TEER values of endothelial monolayer treated with D were 99.63 ± 1.10% (20 × 10^−6^ m) and 101.67 ± 1.41% (40 × 10^−6^ m), and for SA‐D, the values were 102.71 ± 4.72% (0.46 × 10^−6^ m) and 90.61 ± 4.91% (0.69 × 10^−6^ m), respectively. At 40 h, a recovery to 106.99 ± 2.28% for 0.46 × 10^−6^ m SA‐D could be observed (Figure 5B). A long‐term observation with media exchange at 42 h clearly indicated a stable barrier up to 100 h. For SA‐D with higher concentration (0.69 × 10^−6^ m), the TEER dropped to 89.21 ± 4.81% after 20 h but recovered to 102.33 ± 5.70% at 40 h and after media exchange showed an increase to 131.69 ± 6.86% at 100 h. Data of the BBB integrity analysis prove that the dendrons had nearly no effect on in vitro barrier function and SA‐D showed only minor effects, but were fully recovered.

For a successful brain delivery across an intact BBB, ideally, a transcytotic pathway is addressed, i.e., vesicle‐based transport across the interior of a cell.^[^ ^4^ ^]^ The endosomal uptake in brain endothelial cells was investigated to assess if D and SA‐D are transported via the transcytotic brain delivery pathway. Therefore, cellular uptake was first studied in vitro for murine brain endothelial cells (bEnd.3 cells). Stained with an endothelial cell marker CD105 (green), bEnd.3 cells showed a strong and distinct uptake for Cy5‐D and Cy5‐SA‐D (both in red) in cellular vesicles, as shown in Figure 5C. Costaining for early endosomes (EEA) or for late endosomes (LAMP), bEnd.3 cells revealed that after 24 h, Cy5‐D and Cy5‐SA‐D were mainly localized in late endosomes. From late endosomes, either a release into the cytosol, trafficking to lysosome, or trafficking for endosomal release could occur. Colocalization with early and late endosomes showed the uptake of Cy5‐D and Cy5‐SA‐D by the endosomal pathway. A transcytosis mediated transport performance of dendron and SA‐D conjugates, followed by a potential uptake into target cells would be crucial to reduce side effects and of interest for drug delivery study. To address this question, uptake into murine primary glia cells positive for glial fibrillary acidic protein (GFAP) and NeuN positive primary murine neurons was tested. Confocal imaging revealed a very low uptake in GFAP positive astrocytes compared to a strong uptake in NeuN positive neurons. The neurons showed a strong uptake into the cytosol as well as into the dendrites and axons (Figure 5C; Figure S14, Supporting Information). Low magnification images revealed 99 ± 1.5% of Cy5‐D‐positive and 81 ± 1.6% of Cy5‐SA‐D‐positive neurons (Figure S14, Supporting Information). If the observed specific uptake for neurons could be confirmed in vivo, such observed cell type‐specific uptake could be crucial to reduce side effects in other, i.e., nondiseased cells in future clinical applications.

### 2.6 In Vivo Brain Uptake of D and SA‐D

**Figure 6:**
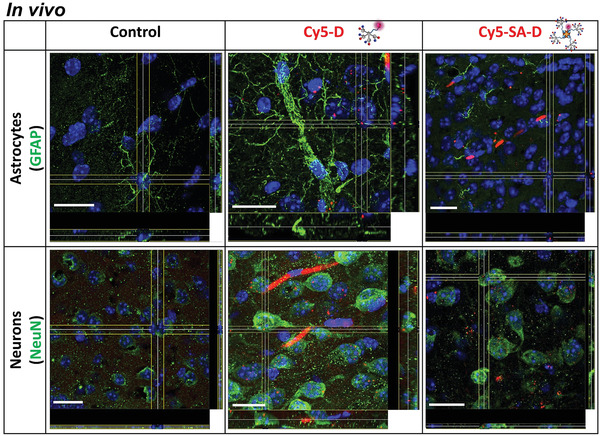
Confocal 3D orthogonal view for Cy5‐D and Cy5‐SA‐D delivery in vivo. Mice were tail intravenous injected with Cy5‐D (red), Cy5‐SA‐D (red), or PBS as control. After 24 h, mice were perfused, brains were sliced and stained with DAPI (blue), GFAP (green), and NeuN (green). The scale is 20 µm. Animated 3D reconstructions can be found in Movies S1–S6 of the Supporting Information.

Next, we have studied the ability of D and SA‐D to cross the BBB and reach the brain in vivo in mice after systemic application (tail intravenous injection). Cy5‐D and Cy5‐SA‐D were used in order to follow brain delivery (**Figure** **6**; Movies S1–S6, Supporting Information) and biodistribution (Figure S15, Supporting Information) by fluorescence microscopy. Mice were treated for 24 with Cy5‐D and Cy5‐SA‐D, and perfused with 4% paraformaldehyde (PFA). Brains were sliced and costained for cell‐specific markers with commercial antibodies. GFAP was used for astrocytes (Figure 6, Movies S1–S3, Supporting Information) and neuronal nuclei (NeuN) were used for neurons (Figure 6; Movies S4 and S6, Supporting Information). Representative orthogonal view and animated 3D reconstruction from *z*‐stacks revealed that distinct strong, vesicular signals for cellular uptake were obtained in neurons positive for NeuN (Figure 6; Movies S5 and S6, Supporting Information). It is worth noting that, very low or no uptake was observed for GFAP positive astrocytes (Figure 6; Movies S2 and S3, Supporting Information). These data demonstrated the successful delivery of the APDs and their bioconjugates into neuronal cells with remarkable selectivity over astrocytes, comparable to the in vitro cell uptake data (Figure 5C). The differences in cellular uptake could be explained by different uptake mechanisms for these different cell types. Transport into astrocytes often proceeds via phagocytosis, pinocytosis as well as endocytosis. Neurons are not considered phagocytic cells, but they reveal a high synaptic uptake and also endocytosis occurs. Since A*β* possesses not only intracellular but also extracellular cytotoxicity, a strong uptake of D or SA‐D into neurons could likely inhibit toxic effects of intracellular A*β* ^[^ ^28^ ^]^ and would reduce D and SA‐D off‐target uptake by the surrounding astrocytes. Both in vitro and in vivo data indicate a great potential of D and SA‐D for future studies in A*β* cytotoxicity inhibition in Alzheimer's disease.

### 2.7 Inhibition of A*β* Toxicity and A*β*–Cell Interactions

**Figure 7:**
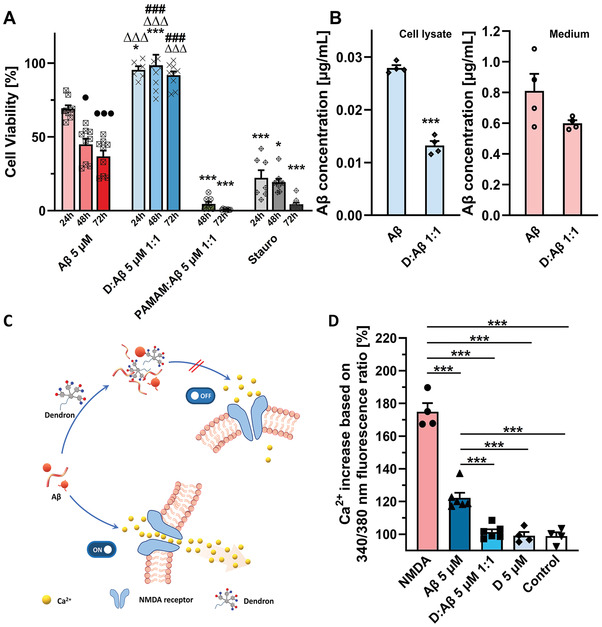
Effects of D on A*β* toxicity and cell interactions. A) Cell viability of primary murine neuronal cells after A*β*, D:A*β* 1:1 (molar ratio), PAMAM (G3):A*β* (equimolar ratio) complexes and respective controls for different incubation times (in all ratios, the A*β* concentration is 5 × 10^−6^ m). Data are presented as mean with SEM. *n* ≥ 6; one‐way ANOVA; *ns* > 0.05, * *p* ≤ 0.05 versus A*β* control of respective time 24, 48, and 72 h, ∆*p* ≤ 0.05 versus staurosporine (Stauro) group of 24, 48, and 72 h, # *p* ≤ 0.05 versus PAMAM:A*β* groups of 48 and 72 h, ● *p* versus A*β* 24 h, ***/###/∆∆∆/●●● *p* ≤ 0.001. B) ELISA assay to quantify the A*β* level in media and cell lysate of bEnd.3 cells with or without D and treatment for 24 h. C) Schematic illustration of A*β*‐induced calcium influx and A*β*‐D interaction to inhibit the neuronal calcium influx and D) calcium Fura‐2 AM relative 340/380 ratio of primary neuronal cells treated with A*β* 5 × 10^−6^ m, equimolar ratios of D and A*β* (A*β* 5 × 10^−6^ m), D (5 × 10^−6^ m), and media (Control). NMDA (1 × 10^−3^ m) was set as positive control for the assay. Data are presented as mean with SEM. For panel B and D, *n* ≥ 4; one‐way ANOVA. *ns* > 0.05, * *p* ≤ 0.05, ** *p* ≤ 0.01, *** *p* ≤ 0.001.

As described above, we have demonstrated that D and SA‐D can effectively inhibit A*β* fibrillation. To verify the potential of D and SA‐D to reduce the toxicity of A*β* fibrils on primary murine neuronal cells, we performed a proof‐of‐concept cell vitality experiment by treating neurons with A*β*‐D/SA‐D mixtures. A*β* fibrils were formed at 37 °C for 16 h on an orbital shaker prior to the cell application. After treatment with A*β* 5 × 10^−6^ m, reduced cell viability of 69.1 ± 2.3% (24 h) 44.9 ± 8.85% (48 h) and 36.8 ± 6.67% (72 h) was observed (**Figure** **7**A). Incubated D with A*β*, both 1:1 and 4:1 (molar ratio, A*β* 5 × 10^−6^ m) showed significant inhibition of neuron cytotoxicity with no significant change within 72 h (Figure 7A; Figure S16, 95.4 ± 2.48% (24 h), 98.4 ± 7.23% (48 h), and 91.7 ± 2.65% (72 h) for D:A*β* 1:1 and 78.8 ± 4.42% (24 h), 88.9 ± 8.07% (48 h), and 79.9 ± 1.96% (72 h) for D:A*β* 4:1). For SA‐D:A*β* 1:1 (equimolar ratio, A*β* 5 × 10^−6^ m), the cell viability also showed high viability of 90.5 ± 7.17% (48 h) and 84.2 ± 5.07% (72 h) over time (Figure S16C, Supporting Information). D itself showed a cell vitality of 91 ± 1.4% (20 × 10^−6^ m) and 80 ± 2.8% (40 × 10^−6^ m), and for SA‐D, a vitality of 97.3 ± 2.7% (2.5 × 10^−6^ m) and 77.8 ± 2.2% (5 × 10^−6^ m) was observed (Figure S16A, Supporting Information). These results highlight the impact of APDs and its bioconjugates (SA‐D) in preventing neuronal death without inducing increase of cell cytotoxicity over time. As control, PAMAM dendrimers (G3, 5 × 10^−6^ m) with their positively charged amino groups display high cytotoxic toward primary neuronal cells (2.1 ± 0.36%, Figure S17, Supporting Information) as well as for intestinal cell line Caco‐2.^[^ ^29^ ^]^ PAMAM:A*β* 1:1 could not inhibit A*β*‐induced cytotoxicity and even further reduced cell viability to 4.56 ± 1.34% (48 h) and 0.764 ± 0.132% (72 h) (Figure 7A). These data demonstrated that incubating equimolar ratios of D with A*β* effectively inhibited the cell toxic effect on primary neuronal cells.

The possible mechanism of D inhibiting A*β* cytotoxicity is evaluated by studying its effect on A*β*–cell interactions. On the one hand, due to the A*β*–dendron interaction, *β*‐sheet formation was significantly suppressed, reducing the amount of subsequent A*β* fibrils, which could further interact with the cell membrane, leading to disruption of the Ca^2+^ homeostasis of neurons.^[^ ^2^, ^30^ ^]^ On the other hand, D‐bound‐A*β* might reduce the binding efficiency of A*β* to membrane proteins, such as nerve growth factor, *N*‐methyl‐d‐aspartate (NMDA) receptor or insulin receptors, which would lead to A*β*‐induced cell stress and trigger the pathologic cascade (Figure 7C).^[^ ^31^ ^]^ The effect of the inhibition on NMDA‐receptor binding was investigated by evaluating A*β*‐induced Ca^2+^ influx in Fura‐2‐AM loaded primary neuronal cells with and without APDs. As shown in Figure 7D, equimolar ratios of D:A*β* significantly inhibit A*β*‐induced Ca^2+^ influx (122 ± 3.07%) to 101 ± 1.67%, to reach the comparable level as the control (98.8 ± 2.31%) or D alone (99.1 ± 2.22%). These data indicate that introducing D could reduce the interactions between A*β* and cells.

Not only cell membrane interaction, but also, the impact of the APDs on A*β* monomer‐cell uptake has been investigated. bEnd.3 cells were incubated with A*β* monomers and D:A*β* (1:1) for 24 h and the A*β* levels were quantified in both cell lysate and media using ELISA (Human A*β*42 Ultrasensitive ELISA kit, Invitrogen, KHB3544). APDs interacting with A*β* significantly reduced their cell uptake from 0.028 ± 0.0005 µg mL^−1^ (A*β* only) to 0.013 ± 0.0008 µg mL^−1^ (D:A*β* 1:1), while the A*β* levels in media with (0.600 ± 0.0195 µg mL^−1^) and without APDs (0.810 ± 0.11 µg mL^−1^) did not change significantly (Figure 7B). Less cellular uptake for D:A*β* could be explained by inhibition of A*β*–cell interactions due to interaction with D as well as a higher rate of A*β* degradation after binding. Such successful inhibition of A*β*‐induced Ca^2+^ influx, reduced cell uptake as well as higher rate of A*β* degradation will protect against A*β*‐mediated cell toxicity. Hence, we postulate that the D‐bound‐A*β* could be an important mechanism to perturb A*β*‐induced cell stress by reducing the A*β*–cell interactions and reduce neuronal dysfunction, which might break the neurodegeneration cascade in Alzheimer's disease.

## 3 Conclusion

We have demonstrated for the first time the superior capability of amphiphilic polyphenylene dendron (APD) and its protein bioconjugates (SA‐D) in reversing amyloid fibrillation and inhibiting A*β*‐induced primary neuronal cell toxicity. Attributed to the alternating negatively charge sulfonic acid and hydrophobic n‐propyl peripheral groups together with the aromatic skeleton, APDs reveal an antiamyloid function in vitro and are transported into the brain in vivo. APDs and SA‐D effectively inhibited full‐length A*β* peptide (1‐42) fibrillation, and they were also able to disassemble already formed A*β* fibrils. More importantly, applying our system significantly reduced the A*β*‐induced cell interactions and toxicity on primary murine neurons. Besides, we demonstrated that APDs and SA‐D showed high biocompatibility to both endothelial cells and primary neurons, and no impact on BBB integrity in vitro. After systemic noninvasive application in vivo, they successfully passed the BBB, and high uptake in neurons compared to astrocytes both in vitro and in vivo was observed. These superior properties compared to other dendritic systems endow APD and SA‐D great potential in future drug development for the treatment of amyloid diseases, and could reduce side effects in nondiseased cells. Moreover, APDs with the dual action of A*β* fibril inhibition/disassembly and reducing A*β* monomer to A*β* fibril toxicity would not be limited to the treatment in early‐stage of AD or in their effect only on A*β* fibrils as many other inhibitors are, which might be one reason why many of them failed in clinical trials.^[^ ^32^ ^]^ Not only APDs but also SA‐D with its four binding pockets of the streptavidin core, offers the possibility to integrate additional functionalities (e.g., tracers and drugs) into our system. We have demonstrated the potential of APDs for antiamyloid strategies and future investigations involving AD animal models will follow to validate their potential in Alzheimer models in vivo. Furthermore, the herein presented SA‐D provides a powerful platform as a multifunctional delivery system to the brain. In a broader context, we envision that our system could also function on other protein‐misfolding‐induced disorders.

## 4 Experimental Section

### Ethics Statement

The experimental procedures were authorized by the ethical committee and the authority “Landesuntersuchungsamt Rheinland‐Pfalz” the protocol number is “Aktenzeichen 23 177‐07/G 16‐1‐024.” Investigations were performed according to the principles of laboratory animal care (European, national and international laws).

### Materials

Reagents were of analytical grade and used as received and purchased from Sigma‐Aldrich (Seelze or Hamburg, Germany), unless otherwise stated. PAMAM dendrimer, generation 3.0 with amino surface group was purchased from Sigma‐Aldrich (412422). Amyloid beta (1‐42) (A*β* (1‐42), DAEFRHDSGYEVHHQKLVFFAEDVGSNKGAIIGLMVGGVVIA) was purchased from Anaspec Incorporated (purity ≥95%). Water was purified by a Milli‐Q filter system.

### Instruments for Measurements

Fluorescence spectra were measured on TECAN. TEM images were recorded on JEOL JEM1400 transmission electron microscope. The absorbance of A*β* solution was measured by Nanodrop 2000 (Thermo Scientific). CD spectra were measured on JASCO J‐1500 Circular Dichroism Spectrophotometer. ATR FT‐IR spectra were measured on Tensor II (Bruker).

### Systemic Delivery of Dendron and Bioconjugates

Cy5‐D (11.2 × 10^−6^ m final concentration in blood) or Cy5‐SA‐D (2.8 × 10^−6^ m final concentration in the blood) were injected via the tail vein in mice. For control, mice got a PBS injection. Perfusion, with PBS supplemented with heparin‐sodium (Ratiopharm, 5000 E.I.) followed by PFA 4%, of the mice was performed 24 h after particles injection. The organs (brain, liver, kidney, spleen, lung, and heart) were collected and stored in PBS at 4 °C.

### Immunohistochemistry

For immunohistochemistry organs were incubated with 30% sucrose for 24 h and frozen in −80 ˚C freezer to obtain 30 µm slices using a freezing microtome (Leica CM 1325). Slices were washed three times with PBS 0.01 m, unmasked with 1% sodium dodecyl sulfate for 5 min at room temperature, blocked and permeabilized with 7% normal donkey serum (017‐000‐121, Dianova, Hamburg) and 0.8% Triton in PBS 0.01 m. First antibody was incubated with 2% bovine serum albumin (001‐000‐161, Dianova, Hamburg) and 0.3% Triton in PBS 0.01 m overnight at room temperature. After three times washing with PBS 0.01 m the secondary antibody was incubated for 2 h at room temperature in 2% bovine serum albumin in PBS 0.01 m. DAPI solution (0.5 µg mL^−1^) was incubated for 10 min. Before mounting with Fluoromount‐G (SouthernBiotech, USA) slices were washed three times with PBS 0.01 m and one time with Milli‐Q water.

### Imaging and Image Preparation

Images were taken by a TCS SP5 confocal (Leica) using the LAS AF software. Projections of *z*‐stacks were visualized using ImageJ. For in vivo confocal images, *z*‐stacks were deconvolved by Huygens Essential software. Animated 3D reconstructions were exported by Imaris software, shown in Movies S1–S6 of the Supporting Information.

### Cell Culture bEnd.3 Cells

A murine cell line, bEnd.3, from brain endothelioma (American Type Culture Collection, Manassas, VA, USA) was cultured as recommended by the manufacturer at a humidified atmosphere with 37 °C and 5% CO_2_.^[^ ^33^ ^]^ DMEM (Glutamax, gibco by life technology, Darmstadt, Germany) supplemented with 10% fetal calf serum and 2% penicillin/streptomycin was the used media. For experiments, passages 10–30 were used with 80 000 cells per transwell insert, or 100 000 cells per IBIDI‐8‐well‐chamber.

### Generation and Culture of Primary Murine Cortical Neuronal Cells

Primary murine neuronal dissociated cell culture were performed as previously described by Kaech and Banker^[^ ^34^ ^]^ with changes according to Beaudoin et al.^[^ ^35^ ^]^ Forebrains of new born mice (postnatal day 1) were isolated. Following meninges depletion the tissue was washed three times with ice‐cold HBSS ‐/‐ and incubated in 0.05% trypsin/EDTA (gibco by life technology, Darmstadt, Germany) for 20 min, followed by incubation with DNase I (Roche, 11284932001) (2000U in 5 mL) for 5 min. Brain tissue was homogenized in Neuronal Plating Medium (10% horse serum, 0.6% glucose) and vital cells were counted (Countess FL cell counter). Primary neurons were seeded in plating medium with cell numbers of 200 000 cells per well of 24‐well plate. The medium was replaced with Neuronal Maintenance Medium Neurobasal Medium (gibco by life technology, Darmstadt, Germany) with 2 × 10^−3^ m glutamine (gibco by life technology, 25030024) and supplemented with B27 Supplement (gibco, 17504044)] after 30 min. Cells were treated with ARAC (5 × 10^−6^ m) on day 2 in vitro. Once a week one‐third of the medium was replaced with fresh Neuronal Maintenance Medium and ARAC treatment was repeated.

### Cell Uptake Study and Immunocytochemistry

Cy5‐D or Cy5‐SA‐D were applied in a final concentration of 0.45 × 10^−6^ and 1.8 × 10^−6^ m to bEnd.3 cells or primary murine neurons for 24 h. Cells were finally fixed with PFA 4%. For immuncytochemisty cells were washed three times with PBS 0.01 m, blocked and permeablized with 7% normal donkey serum (017‐000‐121, Dianova, Hamburg) and 0.8% Triton in PBS 0.01 m. First antibody was incubated with 2% bovine serum albumin (001‐000‐161, Dianova, Hamburg) and 0.3% Triton in PBS 0.01 m overnight at room temperature. After three times washing with PBS 0.01 m the secondary antibody was incubated for 2 h at room temperature in 2% bovine serum albumin in PBS 0.01 m. DAPI solution (0.5 µg mL^−1^) was incubated for 10 min. Before mounting with Fluoromount‐G (SouthernBiotech, USA) slices were washed three times with PBS 0.01 m and one time with Milli‐Q water.

### Preparation of A*β* (1‐42) Stock Film

A*β* (1‐42) stock solution was prepared according to the technical data sheet from the company. A*β* (1‐42) powder (1 mg) was first dissolved in 440 µL HFIP and sonicate for 10 min. Then the stock solution was aliquoted to 0.1 mg per tube and HFIP was removed by rotational vacuum concentrator (RVC 2–18 CDplus, Martin Christ Gefriertrocknungsanlagen GmbH). The stock films were then stored below −20 ˚C.

### A*β* Fibrillation

A*β* (1‐42) stock film (0.1 mg) was dissolved in 2.5 µL DMSO and diluted with 100 µL DPBS solution. The solution was sonicated with ice for 8 min to fully dissolve A*β* (1‐42). The concentration of A*β* (1‐42) monomer solution was confirmed by nanodrop. To obtain A*β* oligomer, A*β* (1‐42) monomer solution was incubated undisturbed at room temperature for 24 h. For fibrils, the monomer solution was *β* incubated at 37 ˚C with vigorously shaking for 16 h. Preformed fibrils (5 × 10^−6^ m) were incubated with D, SA‐D or PAMAM for 16 h in PBS for further investigations.

### Cell Viability Assay Including A*β* Toxicity

Viability for bEnd.3 cells (80 000 cells per well, 50 µL media) and primary neuronal cells (200 000 cells per well, 500 µL media) was investigated using the cell viability assay CellTiter‐Glo Assay (Promega, G7570). After bEnd.3 cells reached 100% confluence, bEnd.3 cells and neuronal cells were treated with D (20 × 10^−6^ or 40 × 10^−6^ m), SA‐D (2.5 × 10^−6^ or 5 × 10^−6^ m), A*β* (5 × 10^−6^ m) or a combination of D:A*β* and SA‐D:A*β* and staurosporine (Stauro, 1 × 10^−6^ m) as control, respectively. A*β* stock film (0.1 mg) was dissolved in 2.5 µL DMSO and diluted with 100 µL DPBS solution. The solution was sonicated with ice for 8 min to fully dissolve A*β*. The concentration of the A*β* monomer solution was confirmed by nanodrop. 250 µL of D or SA‐D mixed with A*β* (10 × 10^−6^ m) with different ratios (D:A*β* = 4:1 and 1:1, SA‐D:A*β* = 1:1) were prepared in neuron media. The mixture was then incubated at 37 ˚C for 16 h in advance to the toxicity assay A*β* alone was also incubate at the same condition to obtain fibrils. Afterward, the A*β* (5 × 10^−6^ m), D:A*β* = 4:1 and 1:1, SA‐D:A*β* = 1:1 was incubated with primary neurons with 5 × 10^−6^ m A*β* for 24, 48, and 72 h. The CellTiter‐Glo Assay was performed according to the manufacturer's instructions. Luminescence was detected using a GloMaxMulti 96‐well plate reader (Promega). Cell Viability in % was related to untreated control cells (100%). All the experiments were run at least two times in triplicates.

### Calcium Fura‐2 AM Assay

Calcium influx in primary neuronal cells was investigated by Fura‐2 AM no Wash Calcium Assay Kit (ab176766, abcam PLC). NMDA was used as positive control for NMDA‐mediated calcium entry induced toxicity study. Primary neuronal cells (200 000 cells per well in 24‐well plate, 500 µL media) were treated with Fura‐2 AM (reconstituted in DMSO) in 1 × assay buffer prepared according to the instructions of the supplier and incubate at 37 ˚C for 1 h. Fluorescence signal at Ex/Em = 340/510 nm and Ex/Em = 380/510 nm for 3 min with the testing interval of 4 s. Neuronal cells were either treated with NMDA (1 × 10^−3^ m), A*β* (5 × 10^−6^ m), D:A*β* (1:1, A*β* 5 × 10^−6^ m) mixture, and D (5 × 10^−6^ m) and then monitor the fluorescence changes at Ex/Em = 340/510 nm and Ex/Em = 380/510 nm for 5 min with the testing interval of 4 s. The fluorescence ratio of 340/380 nm was plotted in % with the mean of 3 min testing interval before sample injection set to 100%. All the experiments were run at least two times in duplicates.

### ELISA Assay

The cellular level of A*β*1‐42 was quantified by Human A*β*42 ultrasensitive ELISA Kit (KHB3544, Invitrogen, Thermo Fisher Scientific). bEnd.3 cells (100 000 cells per well, 500 µL media) were seeded in 24‐well plate and treated with A*β* (5 × 10^−6^ m) or D:A*β* (4:1 and 1:1, A*β* 5 × 10^−6^ m) for 24 h. Then the media was collected from each well and the cells were lysed and centrifuged to get the extractions. The A*β* concentration was tested following the instruction of the ELISA kit. All the samples were run as two independent treatments in duplicates.

### Membrane Impedance Measurement

The measurement of TEER and capacitance of the cell layer in the in vitro BBB model of a bEnd.3 monolayer on a transwell insert was measured by a CellZscope system (NanoAnalytics, Münster, Germany).

### Fibrillation Kinetics

ThT was chosen as the indicator for A*β* (1‐42) aggregation behavior characterization. ThT was dissolved in PBS buffer with a concentration of 2 mm as a stock solution and stored at 4 ˚C. For measurement of ThT fluorescence during the fibrillation process, A*β* (1‐42) was diluted with DPBS into different concentrations and mixed with ThT solution, the final concentration of ThT was 10 × 10^−6^ m. The kinetics was then conducted in 96‐well plates (Greiner μClear, black with clear bottom) with a 16 h period of fibrillation time at 37 ˚C. The fluorescence intensity was tested every 10 min, and with excitation of 450 nm and emission of 490 nm.

For A*β* (1‐42) fibrillation kinetics, HFIP‐treated stock film was dissolved in 2.5 µL DMSO and further diluted with 100 µL PBS. The solution was sonicated for 8 min and vortex for 10 s. The concentration of the solution was calculated by its UV–vis absorbance tested via Nanodrop.

For kinetics of A*β* (1‐42) interacting with D and SA‐D, D or SA‐D was mixed with A*β* and ThT, and further diluted with DPBS to prepare different molar ratios, the final volume of solution is 200 µL with the concentration of ThT 10 × 10^−6^ m. Each condition was performed in triplicate.

For Fmoc‐ISA fibrillation kinetics, PROTEOSTAT protein aggregation assay was use to indicate the fibrillation state. Fmoc‐ISA was first dissolved in DMSO to prepare a stock solution with concentration of 10 mm. Fmoc‐ISA monomer (500 × 10^−6^ m), Fmoc‐ISA:D 1:1 (molar ratio) were mixed with proteostat reagent and dilute the in PBS solution based on assay kit (final concentration of Fmoc‐ISA is 500 × 10^−6^ m and the concentration of proteostat reagent was set based on the instruction). The kinetics was conducted in 96‐well plate (Greiner μClear, black with clear bottom) with a duration of 32 h at 37 ˚C. The fluorescence intensity was tested every 10 min, and with excitation of 550 nm and emission of 600 nm.

### Defibrillation Kinetics

A*β* (1‐42) monomer was first prepared to form fibrils in DPBS with ThT. The solution was then lyophilized and redissolve in MilliQ, containing D/SA‐D at different final molar ratios with A*β* (D:A*β* fibril or SA‐D:A*β* fibril) in 96‐well plate. The finial volume for each well is 200 µL, and the concentration of ThT is 10 × 10^−6^ m. The kinetics was then conducted with 16 h at 37 ˚C. The fluorescence intensity was tested every 10 min, and with excitation of 450 nm and emission of 490 nm.

For Fmoc‐ISA defibrillation kinetics, Fmoc‐ISA monomer (final concentration of 500 µm) was first monitored to form fibril through the same kinetics conditions for Fmoc‐ISA monomer and Fmoc‐ISA:D mixture as described above. After 16 h of reaction, the formed Fmoc‐ISA fibrils were treated with D with 1:1 molar ration and continue the kinetics till 32 h. The kinetics was conducted in 96‐well plate (Greiner μClear, black with clear bottom) at 37 ˚C, and the fluorescence intensity was tested every 10 min, and with excitation of 550 nm and emission of 600 nm.

### Transmission Electron Microscopy

10 µL of the sample was added onto the carbon coated‐copper grid for 10 min and the redundant sample was removed with filter paper. The sample was then stained with 4% (w/v) uranyl acetate for 60 s and dried with filter paper. The copper grids were washed with MiliQ for three times to remove the excess uranyl acetate and dried under room temperature.

### CD Spectroscopy

200 µL of the sample was added to a 0.1 cm quartz for far‐UV (180–260 nm) measurements. The bandwidth was 0.2 nm. The scanning speed was 100 nm min^−1^ with a response time of 4 s. Each spectrum was tested for three scans.

### ATR FT‐IR Measurement

Samples (A*β* fibrils, D:A*β* fibrils with different ratios, PAMAM:A*β* fibrils with different ratios) were first prepared in 96‐well plate as the same condition for kinetics experiments. The solution was subsequently lyophilized. ATR FT‐IR spectra were characterized by Tensor II spectrometer (Bruker) equipped with a diamond crystal as ATR element. Each spectrum was an average of 64 scans.

### Statistics

To analyze data for cell viability luminescence of control cells were set to 100%. To compare the calcium influx the fluorescence ratio of 340/380 nm was plotted in %, the mean of 3 min testing interval before sample injection was set to 100%. Quantification of A*β* was done as described in the ELISA user manual by an A*β* standard. Data are presented as mean ± standard error of the mean (SEM). All statistical tests were performed using Prism8.3 (GraphPad Prism, La Jolla, CA). The effects of the different treatments were analyzed by one‐way analysis of variance (ANOVA) followed by Tukey´s multiple comparisons test for post hoc test. Significance was considered at *p* values of * *p* ≤ 0.05, ** *p* ≤ 0.01, *** *p* ≤ 0.001. Confidence Intervals for the data from Figures 5A and 7A,D can be found in Section S3.3 of the Supporting information.

## Conflict of Interest

The authors declare no conflict of interest.

## Supporting information

Supporting Information

Supplementary MovieS1

Supplementary MovieS2

Supplementary MovieS3

Supplementary MovieS4

Supplementary MovieS5

Supplementary MovieS6

## Data Availability

The data that support the findings of this study are available in the Supporting Information of this article.
