# Supplementary material for: Reversing Aβ Fibrillation and Inhibiting Aβ Primary Neuronal Cell Toxicity Using Amphiphilic Polyphenylene Dendrons
Source: Adv Healthc Mater. 2021 Nov 19;11(2):2101854. doi: 10.1002/adhm.202101854 (PMC11468574; doi:10.1002/adhm.202101854)
Supplement: Supplementary file 1 — Supporting Information [file ADHM-11-2101854-s005.pdf]

WILEY-VCH

ADVANCED HEALTHCARE MATERIALS

Supporting Information

for *Adv. Healthcare Mater.*, DOI: 10.1002/adhm.202101854

Supporting Information

Reversing Aβ Fibrillation and Inhibiting Aβ Primary Neuronal Cell Toxicity using Amphiphilic Polyphenylene Dendrons

Siyuan Xiang,† Jessica Wagner,† Thorsten Lückerath, Klaus Müllen, David Y. W. Ng, Jana Hedrich, and Tanja Weil**

Table of Contents

1. Synthesis 3
1.1 Materials 3
1.2 Instruments 3
1.3 Dendron Synthesis 3
1.4 Preparation of Streptavidin (SA)-Conjugates 6
1.4.1 Cy5-labeled SA (Cy5-SA) 6
1.4.2 Preparation of **SA-D** 7
1.4.3 Preparation of SA-B as reference 8
1.5 Characterization of the **SA-D** conjugates 8
1.5.1 Determination of the SA concentration 8
1.5.2 HABA-Assay 9
1.5.3 SDS-PAGE 11
1.5.4 Agarose Gel Electrophoresis 11
1.5.5 Atomic Force Microscopy (AFM) 12
1.5.6 Dynamic Light Scattering 12
2. Anti-amyloid fibrillation behavior 14
2.1 Kinetics study 14
2.1.1 Kinetics of Aβ fibrillation 14
2.1.2 Kinetics of Aβ fibrillation with **D** and **SA-D** at different Aβ concentration 15
2.1.3 Kinetics of Aβ fibrillation with **SA-B** 15
2.1.4 Defibrillation behavior of **SA-D** on Aβ fibril 16
2.2 Mechanism of dendrons in inhibiting amyloid-like fibrillation and disassembly of already formed fibrils 17
2.3 Defibrillation behavior of PAMAM (G3) on Aβ fibril 18
3. Biological Characterization 19
3.1 *In vitro* cell uptake for primary neuronal cells 19
3.2 *In vivo* Biodistribution 19
3.3 Confidence Intervals for the data from Figure 5A, 7A and 7D 20
3.4 Primary neuronal cytotoxicity study of **D** and **SA-D** with Aβ 20
3.5 Cytotoxicity of PAMAM dendrimer (G3) on primary murine neuronal cells 22
4. Movies S1-S6 22

1. Synthesis

1.1 Materials

Organic solvents for reactions and purification were purchased from Fisher Scientific or Acros Organics and used without any further purification (HPLC grade). Ultrapure water for reactions and purifications were obtained from a Merck MilliQ water purification system. All chemicals were purchased from commercial suppliers like Sigma Aldrich, Fisher Scientific, Acros Organics, Thermo Scientific etc. if not stated otherwise and were used without any further purification. For size-exclusion chromatography Sephadex® G25 or G50 in ultrapure water were used.

1.2 Instruments

^{1}H-NMR and ^{13}C-NMR spectra were measured on an Avance III 700 MHz spectrometer in DMSO-d6 at 298 K. ^{13}C-NMR spectra were recorded in j-modulated spin-echo (JMOD) mode. MestReNova was used to analyze the spectra. MALDI-TOF measurements were performed on a Bruker rapifleX MALDI-TOF/TOF and a Waters MALDI Synapt G2-SI. mMass was used for data processing. Absorption spectra and intensities were measured on a SPARK 20M microplate reader from TECAN Group Ltd. The samples were measured in a Greiner 384 flat transparent well plate.

1.3 Dendron Synthesis

Dendron-conjugates **1**, **2** and **3** were synthesized as previously reported. [13]

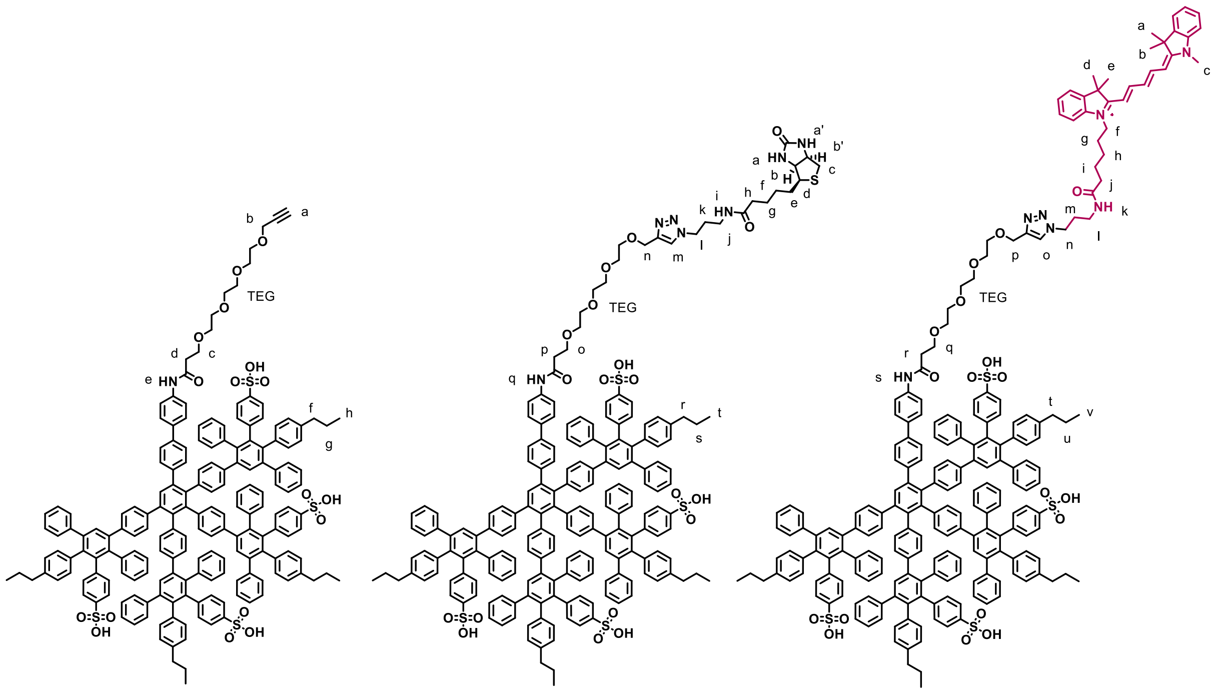

Figure S1. Synthesized dendron-conjugates: unfunctionalized dendron (**D**, **1**), **biotin-D** (**2**) and **Cy5-D** (**3**).

Dendron (**D, 1**) was obtained in 98% yield. All spectral data are in agreement with the literature.[13] ^{1}H NMR (500 MHz, DMSO-d_6): δ(ppm) = 10.03 (s, 1H, H_e), 7.75–7.56 (m, 4H, H_{arom}), 7.50–7.23 (m, 7H, H_{arom}), 7.22–6.54 (m, 84H, H_{arom}), 6.52–6.30 (m, 6H, H_{arom}), 4.11 (m, 2H, H_f), 3.71 (t, J = 5.6 Hz, 2H, H_c), 3.55–3.44 (m, 12H, H_{TEG}), 2.57 (t, J = 4.9 Hz, 2H, H_d), 2.44–2.23 (m, 9H, $H_{a,}$ H_f), 1.47–1.31 (m, 8H, H_g), 0.74–0.61 (m, 12H, H_h).

^{13}C NMR (126 MHz, DMSO): δ (ppm) = 145.54–119.32, 77.08, 69.75, 69.69, 69.47, 68.50, 66.67, 57.47, 36.44, 23.52, 13.11.

MALDI-TOF: *m/z* calcd. for $C_{186}H_{153}NO_{17}S_4$ 2800.00, found 2801.28 $[M+H]^+$, 2824.35 $[M+Na]^+$, 2840.31 $[M+K]^+$, 2862.25 $[M+NaK]^{2+}$, 2878.33 $[M+2K]^{2+}$.

Biotin-D (**2**) was obtained in 97% yield. All spectral data are in agreement with literature.[13]

^{1}H NMR (700 MHz, DMSO-d_6) δ(ppm) = 10.03 (s, 1H, H_q), 8.08 (d, J = 3.0 Hz, 1H, H_{arom}), 7.88 (t, J = 4.5 Hz, 1H, H_i), 7.73–7.66 (m, 2H, H_{arom}), 7.60 (s, 2H, H_{arom}), 7.48–6.27 (m, 100H, H_{arom}, H_a, $H_{a'}$, H_m), 4.49 (s, 2H, H_n), 4.32 (t, J = 7.0 Hz, 2H, H_l), 4.28 (t, J = 6.5 Hz, 1H, $H_{b'}$),

4.13–4.09 (m, 1H, H_b), 3.71 (t, J = 6.2 Hz, 2H, H_o), 3.58–3.44 (m, 12H, H_{PEG}), 3.11–3.06 (m, 1H, H_d), 3.02 (q, J = 6.4 Hz, 2H, H_j), 2.79 (dd, J = 12.5, 5.1 Hz, 1H,H_c), 2.64–2.53 (m, 3H, $H_{c'}$, H_p), 2.45–2.22 (m, 8H, H_r), 2.06 (t, J = 7.5 Hz, 2H, H_h), 1.92 (p, J = 6.7 Hz, 2H, H_k), 1.68–1.23 (m, 14H, H_e, H_f, H_g, H_s), 0.74–0.62 (m, 12H, H_t).

^{13}C NMR (176 MHz, DMSO) δ(ppm) = 172.19, 162.68, 145.51-119.31, 69.76, 69.66, 68.95, 66.64, 63.53, 61.02, 59.19, 55.39, 47.15, 40.02,37.23, 36.44, 35.64, 35.17, 33.90, 33.60, 29.95, 28.19, 28.02, 25.23, 23.52, 13.12.

MALDI-TOF: *m/z* calcd. for $C_{199}H_{175}N_7O_{19}S_5$ 3126.15, found 3149.65 $[M+Na]^+$, 3171.62 $[M+2Na]^{2+}$, 3187.58 $[M+K]^+$, 3193.61 $[M+3Na]^{3+}$.

Cy5-D (**3**) was obtained in 86% yield. All spectral data are in agreement with literature.[13] ^{1}H NMR (700 MHz, DMSO-d_6) δ(ppm) = 10.00 (s, 1H, H_s), 8.35–8.23 (m, 2H, H_{arom}), 7.89–7.81 (m, 1H, H_k), 7.65 (d, *J* = 7.4 Hz, 2H, H_{arom}), 7.53 (dd, *J* = 14.7, 7.4 Hz, 2H, H_{arom}), 7.43–6.15 (m, 117H, H_{arom}), 4.45 (s, 2H, H_p), 4.26 (t, *J* = 7.0 Hz, 2H, H_n), 4.05–3.96 (m, 2H, H_f), 3.70–3.64 (m, 2H, H_r), 3.54–3.40 (m, 15H, $H_{TEG,}$ H_c), 2.98–2.93 (m, 2H, H_l), 2.35–2.22 (m, 8H, H_t), 2.00 (t, *J* = 7.1 Hz, 2H, H_j), 1.84 (q, *J* = 7.0 Hz, 2H, H_m), 1.64–1.54 (m, 12H, $H_{a,}$ $H_{b,}$ $H_{d,}$ H_e), 1.52–1.44 (m, 2H, H_i), 1.43–1.16 (m, 12H, $H_{g,}$ $H_{h,}$ H_u), 0.72–0.54 (m, 12H, H_v).

^{13}C NMR (176 MHz, DMSO) δ(ppm) = 154.33, 145.52–124.18, 123.88, 122.29, 118.68, 111.00, 69.80, 69.72, 68.50, 65.75, 63.57, 48.85, 47.15, 36.55, 36.45, 35.64, 34.40, 29.93, 27.11, 26.94, 24.84, 23.54, 13.14, 12.88.

MALDI-TOF: *m/z* calcd. for $C_{221}H_{198}N_7O_{18}S_4^+$ 3365.37, found 3365.28 $[M]^{\bullet+}$, 3387.26 $[M+Na]^+$, 3410.25 $[M+2Na]^{2+}$.

1.4 Preparation of Streptavidin (SA)-Conjugates

1.4.1 Cy5-labeled SA (Cy5-SA)

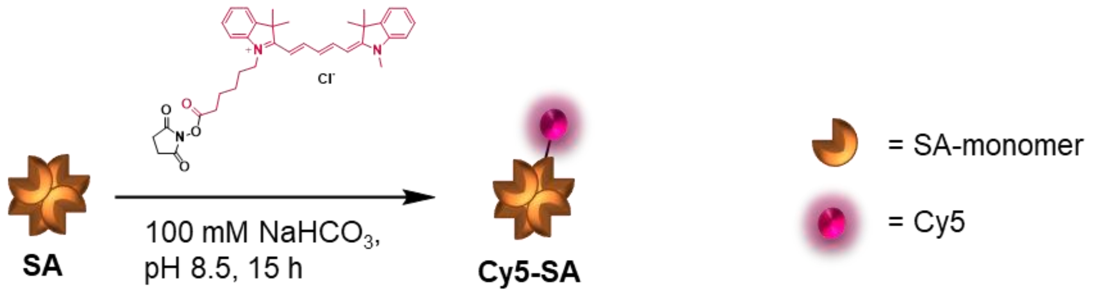

Figure S2. Modification of SA with the fluorophore Cy5. 1 molar equivalent of SA-tetramer was labeled with 1 molar equivalent of Cy5 resulting in 3 unlabeled SA-monomers and 1 labelled SA-monomer statistically.

SA (8 mg, 0.15 µmol) was dissolved in 1 mL 100 mM $NaHCO_3$ buffer, pH 8.5 and Cy5-NHS ester (93 µg, 0.15 µmol) dissolved in 15 µL DMSO was added. The reaction mixture was shaken at room temperature for 15 h in the dark. To remove unreacted Cy5, the reaction mixture was purified using Sephadex G-50 in ultrapure water. After lyophilization Cy5-labelled SA was obtained as a blue solid (7.8 mg, 99%).

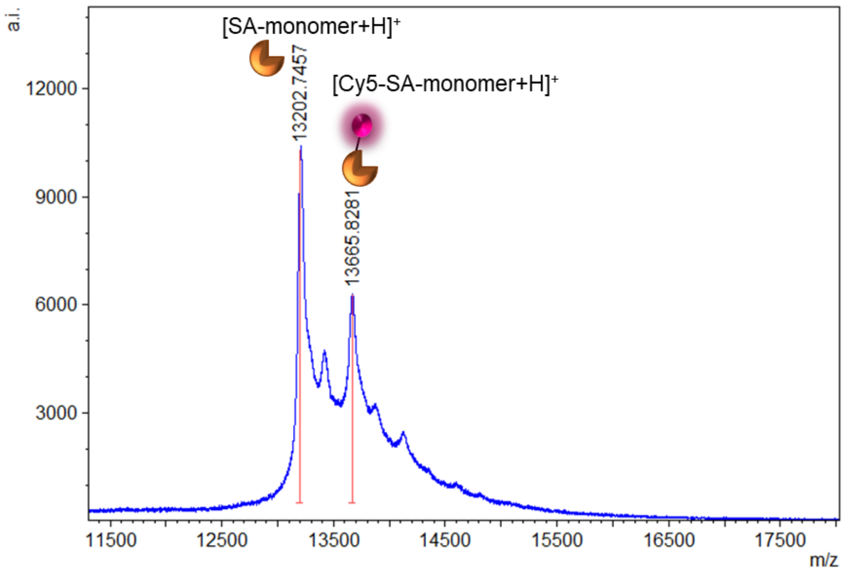

Figure S3. MALDI-TOF mass spectrum of Cy5-labeled SA. Both unlabeled (m/z = 13202.75 [SA-monomer+H]+) and Cy5-labeled SA monomer (m/z = 13665.83 [Cy5-SA-monomer+H]+) were observed due to the statistic labeling with 1 equivalent Cy5 to 1 equivalent of SA-tetramer.

1.4.2 Preparation of **SA-D**

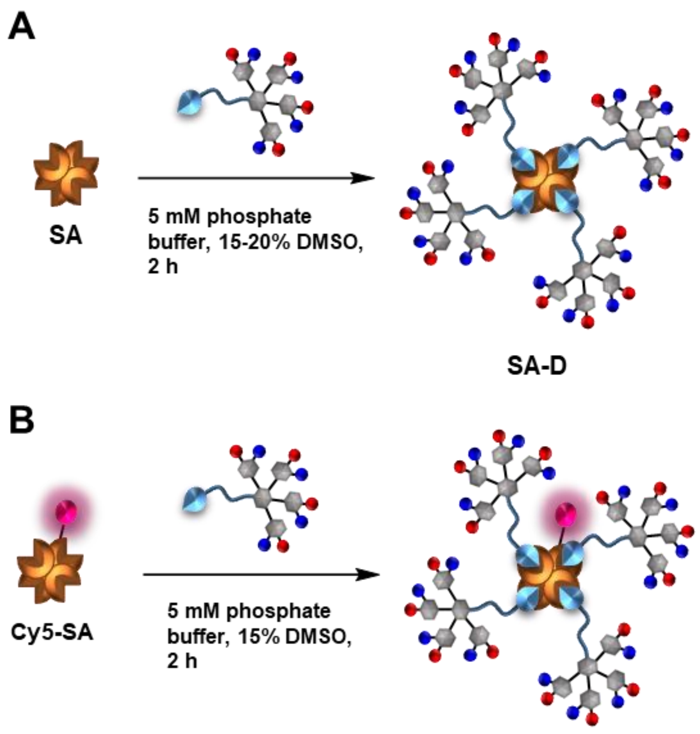

Figure S4. Preparation of (A) dendronized SA (**SA-D**) and (B) dendronized Cy5-SA (**Cy5-SA-D**)

A) Unlabeled **SA-D**:

1.) 2 mg scale: **Biotin-D** (**2**) (522 µg, 167 nmol, 4.5 equiv) was dissolved in 200 µL DMSO and 200 µL autoclaved ultrapure water was added. After vigorous shaking, dendron **2** was added to a solution of SA (2.00 mg, 37.9 nmol) in 600 µL ultrapure water. 10 mg mL^{-1} SA contained 20 mm phosphate buffer pH 6.5 resulting in a ~4 mM phosphate buffer concentration in the mixture. The mixture was gently shaken for 2 h at room temperature and purified by size exclusion chromatography using autoclaved Sephadex G-50 in ultrapure water. The purification was performed under sterile conditions. The concentration was determined by BCA-Assay.

2.) 1 mg scale: For smaller scales, the complex formation was performed at lower concentrations as described for the Cy5-labeled **SA-D** in procedure B.

B) Cy5-labeled SA-D (**Cy5-SA-D**): **Biotin-D** (**2**) (267 µg, 85.1 nmol, 4.5 equiv) was dissolved in 125 µL DMSO and 200 µL 5 mM phosphate buffer was added. After vigorous

shaking, dendron **2** was added to a solution of SA (1.00 mg, 1.89 nmol) in 500 µL 5 mM phosphate buffer, pH 7.4. The mixture was gently shaken for 2 h at room temperature and purified by size exclusion chromatography using autoclaved Sephadex G-50 in ultrapure water. The purification was performed under sterile conditions. The concentration was determined by linear calibration against **Cy5-SA** at a wavelength of λ_{Abs}= 650 nm.

1.4.3 Preparation of SA-B as reference

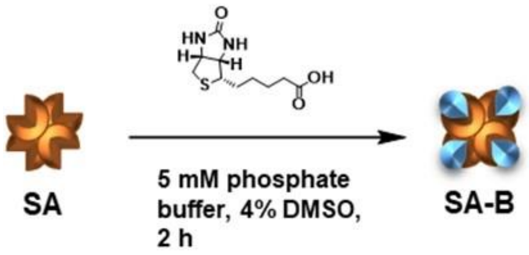

Figure S5. Preparation of SA complexed with free d-biotin (**SA-B**) as reference.

10.4 µL of a 1 mg mL^{-1} solution of d-biotin in DMSO (10.4 µg, 42.5 nmol, 4.5 equiv) were added to a solution of SA (0.5 mg, 9.46 nmol) in 250 µL 5 mm phosphate buffer, pH 7.4. Afterwards, the mixture was treated the same as described for **SA-D** (see above).

1.5 Characterization of the **SA-D** conjugates

1.5.1 Determination of the SA concentration

BCA-Assay for unlabeled samples: The concentration of **SA-D** and **SA-B** was determined by the bicinchoninic acid assay (BCA-Assay) with bovine serum albumin (BSA) as standard for the calibration curve. For this purpose, Pierce™ BCA Protein Assay Kit from Thermo Scientific™ was used and the concentration was determined following the supplier's instructions. BSA standards were used at the concentrations of 0.5, 1, 2.5, 5, 10, 20, 40 and 200 µg mL^{-1}. **SA-D** and **SA-B** conjugates were diluted to 1:100 (v/v) in order to achieve a

concentration that fits to the range of the calibration curve. Both samples and standard were incubated for 30 min at 37 °C with the BCA Assay reagents. Then, the absorption intensity was measured at 562 nm in a 384 flat transparent well plate in triplicates.

Absorbance intensity of Cy5 for labeled samples: The concentration of Cy5-labeled SA was determined using a standard calibration curve with **Cy5-SA** as standard at the concentrations of 1, 5, 10, 50 and 100 µM in MilliQ water. 30 µL of each standard (**Cy5-SA**) and purified **Cy5-SA-D** were transferred in triplicates to a Greiner 384 flat transparent well plate. The absorption intensity was measured at 650 nm.

1.5.2 HABA-Assay

Native SA, purified **SA-B** or **SA-D** solutions were diluted in ultrapure water to afford 1 mg mL^{-1} SA solutions. 25 µL SA solutions were mixed with 0.5 µL 2-(4'hydroxyazobenzene)-benzoic acid (HABA) solution (1 mg mL^{-1} in DMSO) and absorbance spectra (250–750 nm) were measured in a UV-star flat bottom 384-well plate.

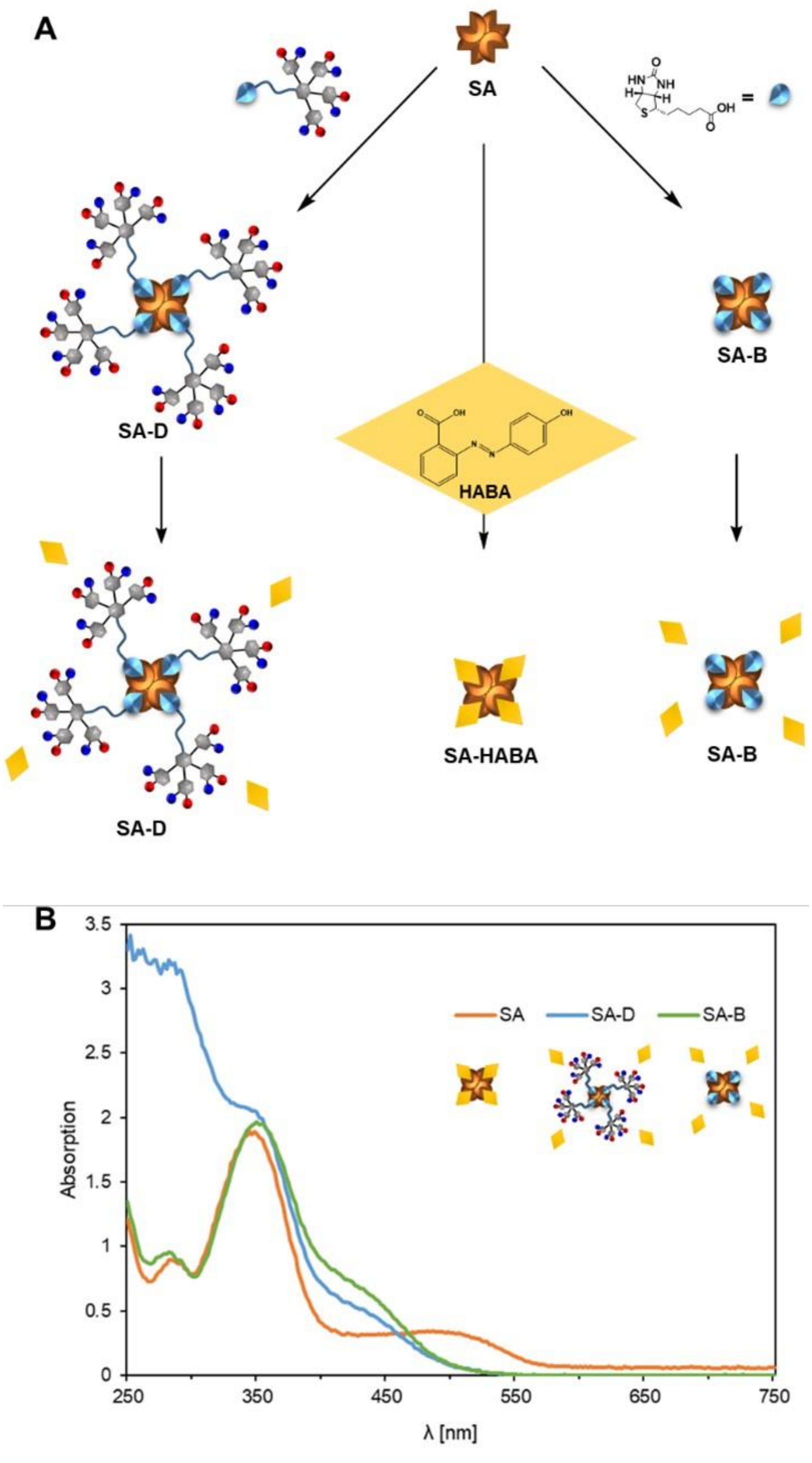

Figure S6. HABA-Assay. (A) Illustration of experimental procedure. (B) Absorbance spectra of SA, **SA-B** and **SA-D** treated with HABA. **Biotin-D** (**2**) binds to SA and thus occupies the biotin-binding sites. HABA binds to the same binding site as d-biotin resulting in an absorption band at 500 nm. Upon binding of d-biotin and biotin-dendron, HABA cannot bind which does not result in an absorption maximum at 500 nm.

1.5.3 SDS-PAGE

SDS-PAGE was performed using a NuPAGE Bis-Tris Gel (4–12%) from Invitrogen. Proteins were applied under (a) denaturing conditions (with heating and DTT) as well as (b) non-denaturing conditions (without heating, no DTT). (a) For denaturing conditions 16 µL of a 0.4 mg mL^{-1} protein solution were mixed with 6 µL sample buffer (NuPAGE, Invitrogen) and 2 µL of 1 m DTT solution and incubated at 95 °C for 10 min. The mixture (20 µL) was loaded on the SDS gel. (b) For non-denaturing conditions 16 µL of a 0.4 mg mL^{-1} protein solution were mixed with 6 µL sample buffer (NuPAGE, Invitrogen) and 2 µL of ultrapure water and the resulting 20 µL solution was loaded to the SDS-Gel. As a reference, 2 µL of Protein Marker VI (10-245) was used. The gel was run in 1× 2-(N-morpholino)ethanesulfonic acid (MES) SDS running buffer with constant voltage of 150 V for 45 min. The gel was washed three times with ultrapure water and stained with Imperial™ Protein Stain from Thermo Scientific for 1 h under gentle orbital shaking. The gel was destained overnight in ultrapure water.

1.5.4 Agarose Gel Electrophoresis

Agarose gel electrophoresis was conducted applying 1% agarose gels based on tris-acetate-EDTA (TAE) buffer. 500 mg agarose was heated up in 50 mL 1× tris-acetate-EDTA (TAE) buffer until complete dissolving of the agarose. The agarose was let set in an agarose gel-mold for 30 min, transferred to an electrophoresis tank and was covered with 1× TAE buffer. 15 µL of each SA (0.4 mg mL^{-1}), **SA-B** (0.4 mg mL^{-1}) and **SA-D** (0.3 mg mL^{-1}) were mixed with 15 µL glycerol and 25 µL of each sample was loaded to the agarose gel loading wells. The electrophoresis was performed in a Mini-Sub® Cell GT from Bio-Rad at 150 V for 45 min on ice. The gel was washed for three times with ultrapure water and stained with Imperial™ Protein Stain from Thermo Scientific for 1 h under gentle orbital shaking. The gel was destained overnight in ultrapure water.

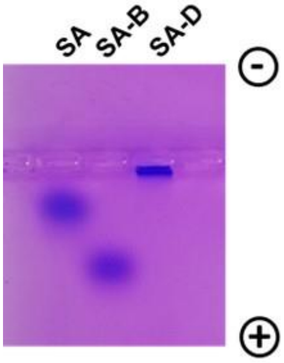

Figure S7. Agarose gel of SA conjugates. SA, **SA-B** and **SA-D** were applied to an agarose gel and the electrophoresis was run at 150 V for 45 min.

1.5.5 Atomic Force Microscopy (AFM)

AFM measurements were conducted on a Dimension FastScan Bio atomic force microscope from Bruker, which was operated in the PeakForce mode. AFM probes with a nominal spring constant of 0.25 N m^{-1} were employed (FastScan-D, Bruker) for measurement in liquid. A circular mica disc (15 mm) was used as the substrate. Measurements were performed at scan rates between 0.8 and 2 Hz. Different areas of the mica substrate were scanned in order to ensure the integrity of the shown images. The images were finally processed by the software NanoScope Analysis 1.8. For sample preparation, the initial **SA-B** and **SA-D** solutions (25 µM in ultrapure water (MilliQ)) were diluted to 600 nM with MilliQ water and subsequently applied onto the freshly cleaved mica substrate. The solution was left to incubate for 15 min in order to deposit the desired species on the mica substrate. After successful adsorption, the supernatant was removed and fresh MilliQ water (250 µL) was added for the measurement. Images were processed using NanoScope Analysis 1.8.

1.5.6 Dynamic Light Scattering

SA-D samples were purified via size exclusion chromatography as described in Section 1.4.2. After determination of the protein concentration by BCA-Assay (Section 1.5.1.1) the sample was diluted with the respective buffer concentrations to achieve an **SA-D** concentration of 0.5 mg mL^{-1} in ultrapure water as well as 25, 50 and 100 mM phosphate buffer, pH 7.4.

Samples were filtered through a 0.2 µm filter prior to the measurement to remove dust particles.

Light scattering measurements were performed on an ALV spectrometer consisting of a goniometer and an ALV-5004 multiple-tau full-digital correlator (320 channels) which allows measurements over an angular range from 30° to 150°. A He-Ne Laser (wavelength of 632.8 nm) is used as light source. For temperature-controlled measurements the light scattering instrument is equipped with a thermostat from Julabo.

Measurements were performed at 20 °C at 9 angles ranging from 30° to 150°.

Table S1. Hydrodynamic radius (R_h) and PDI of **SA-D** in ultrapure water (MilliQ) and 25 mM, 50 mM and 100 mM phosphate buffer. A significant size increase of **SA-D** compared to the control **SA-B** was observed in all buffer concentrations.

	**R_h/nm**	**PDI**
**SA-D** in MilliQ	37.3	0.181
**SA-D** in 25 mm PB	25.1	0.351
**SA-D** in 50 mm PB	23.8	0.208
**SA-D** in 100 mm PB	32.8	0.178
**SA-B** in MilliQ	-	-
**SA-B** in 25 mm PB	3.3[a)]	0.570[a)]

[a)] A bimodal distribution was observed due to a second process at about 300 nm.

2. Anti-amyloid fibrillation behavior

2.1 Kinetics study

2.1.1 Kinetics of Aβ fibrillation

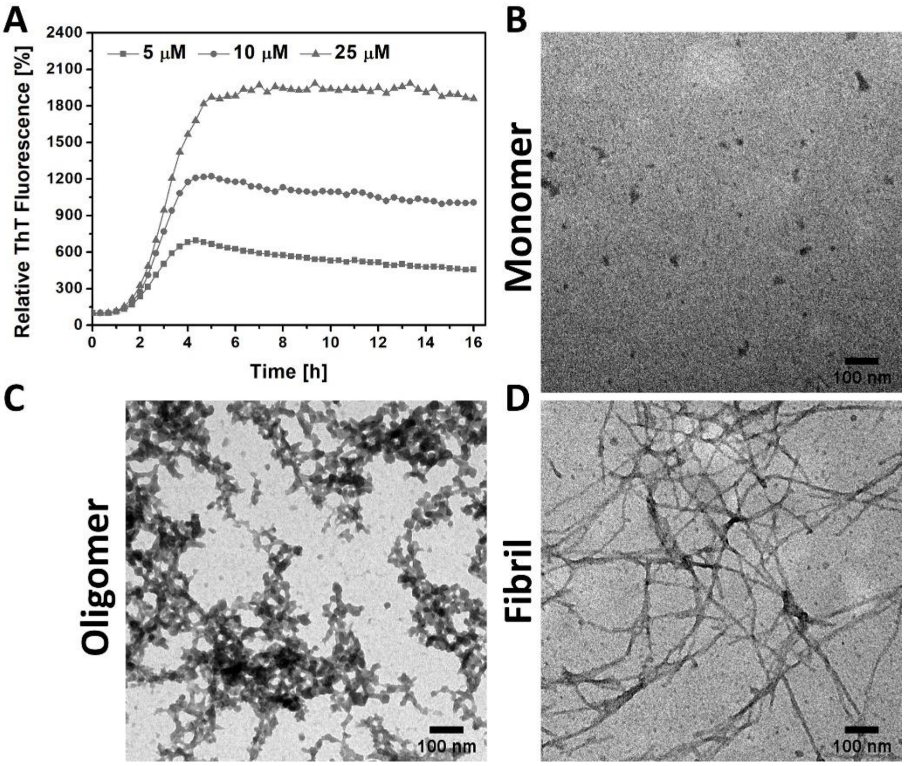

Figure S8. Aβ fibrillation. (A) ThT kinetics of Aβ at a concentration of 5 μM, 10 μM, and 25 μM. Increasing fluorescence indicates the fibrillation of Aβ. (B, C and D) TEM images of Aβ monomers, oligomers, and fibrils. The scale bar is 100 nm.

2.1.2 Kinetics of Aβ fibrillation with **D** and **SA-D** at different Aβ concentration

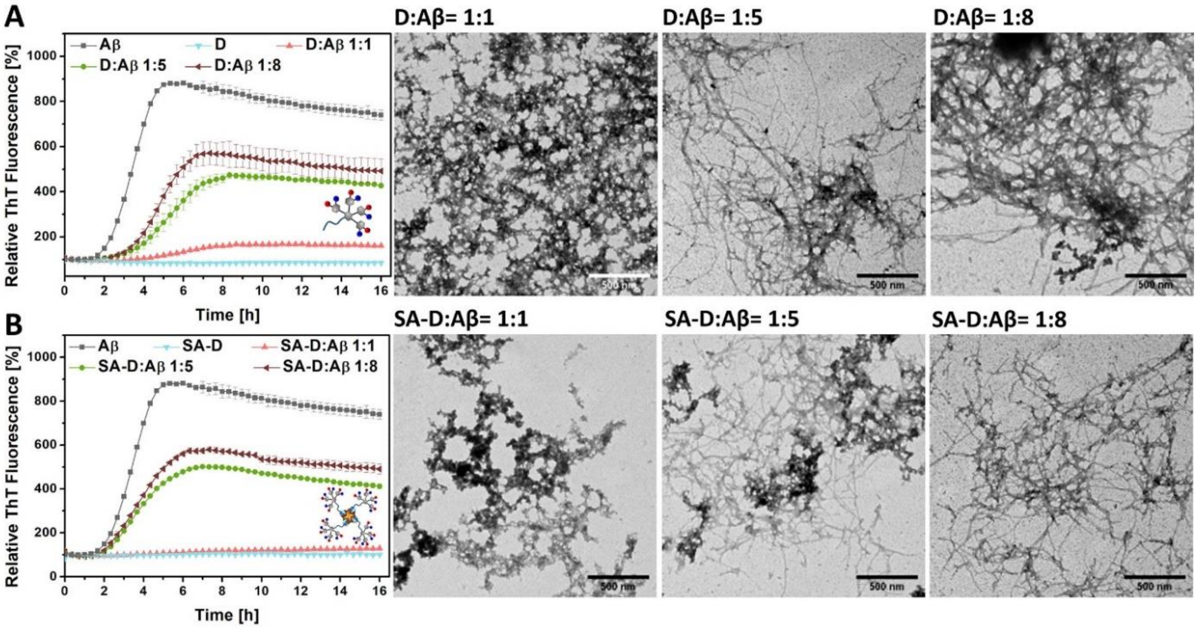

Figure S9. Aβ fibrillation with D and SA-D at increasing Aβ concentrations**.** ThT kinetics of Aβ and **D** (A) and **SA-D** (B) at different molar ratios (Aβ alone, **D**:Aβ = 1:1, 1:5, 1:8 and **SA-D**:Aβ = 1:1, 1:5, 1:8); the concentration of **D** and **SA-D** is 1.6 μM), and the corresponding TEM images of these combinations. The scale bar is 500 nm.

2.1.3 Kinetics of Aβ fibrillation with **SA-B**

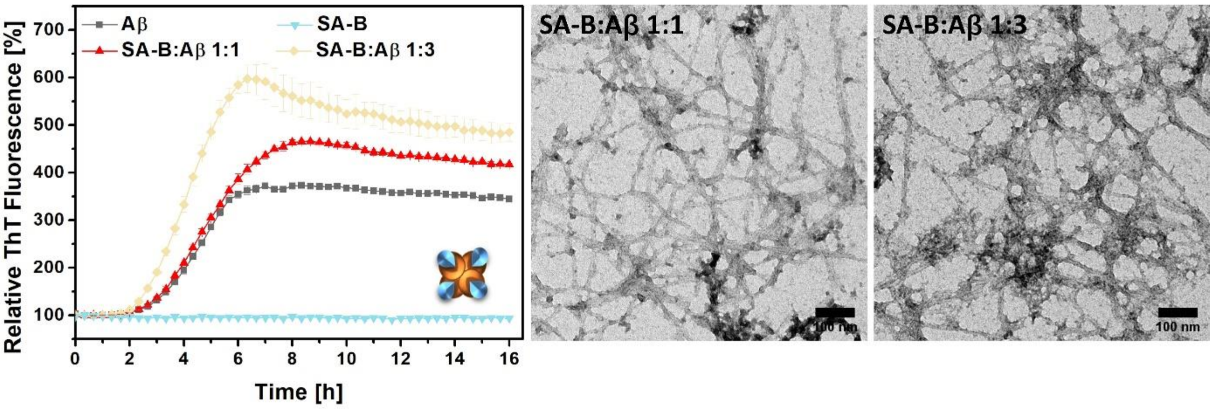

Figure S10. ThT kinetics of Aβ with **SA-B** in 1:1 and 1:3 molar ratio (in all ratios, the Aβ concentration is 5 μM), and the corresponding TEM images of the mixture. The scale bar is 100 nm.

2.1.4 Defibrillation behavior of **SA-D** on Aβ fibril

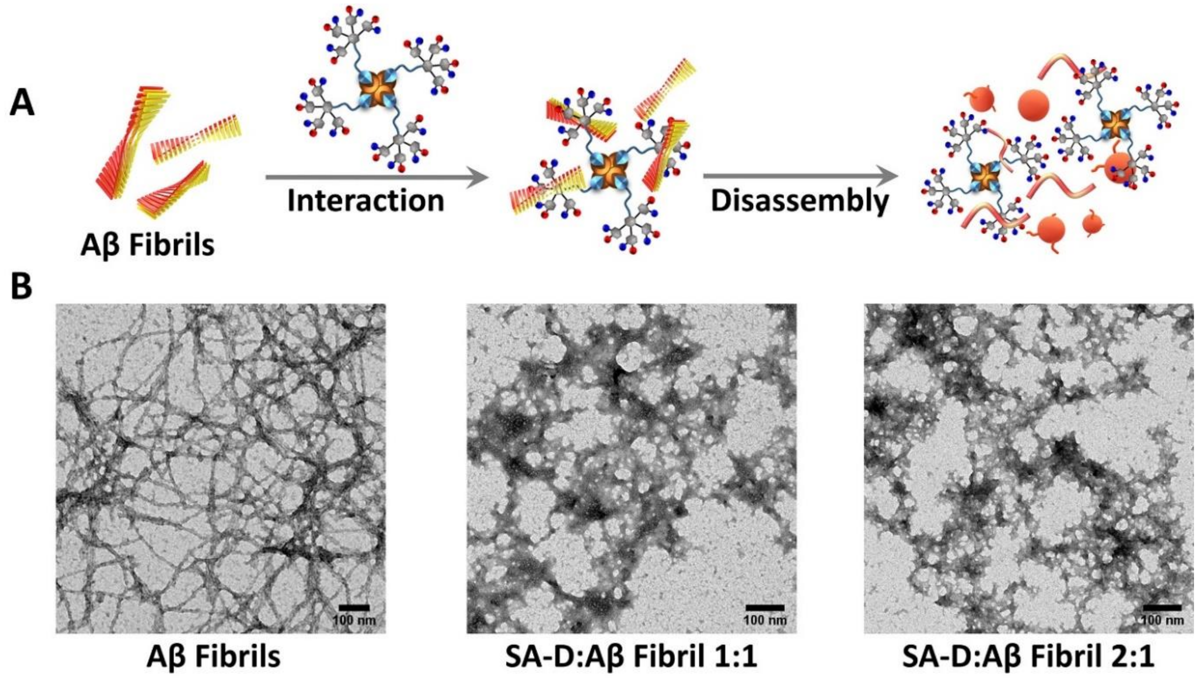

Figure S11. Disassembly performance of SA-D on Aβ fibril. (A) Schematic illustration of **SA-D** disassembling the already formed Aβ fibril, and (B) TEM images of the preformed Aβ fibrils; Aβ fibril mixed with **SA-D** at different molar ratios. The scale bar is 100 nm.

2.2 Mechanism of dendrons in inhibiting amyloid-like fibrillation and disassembly of already formed fibrils

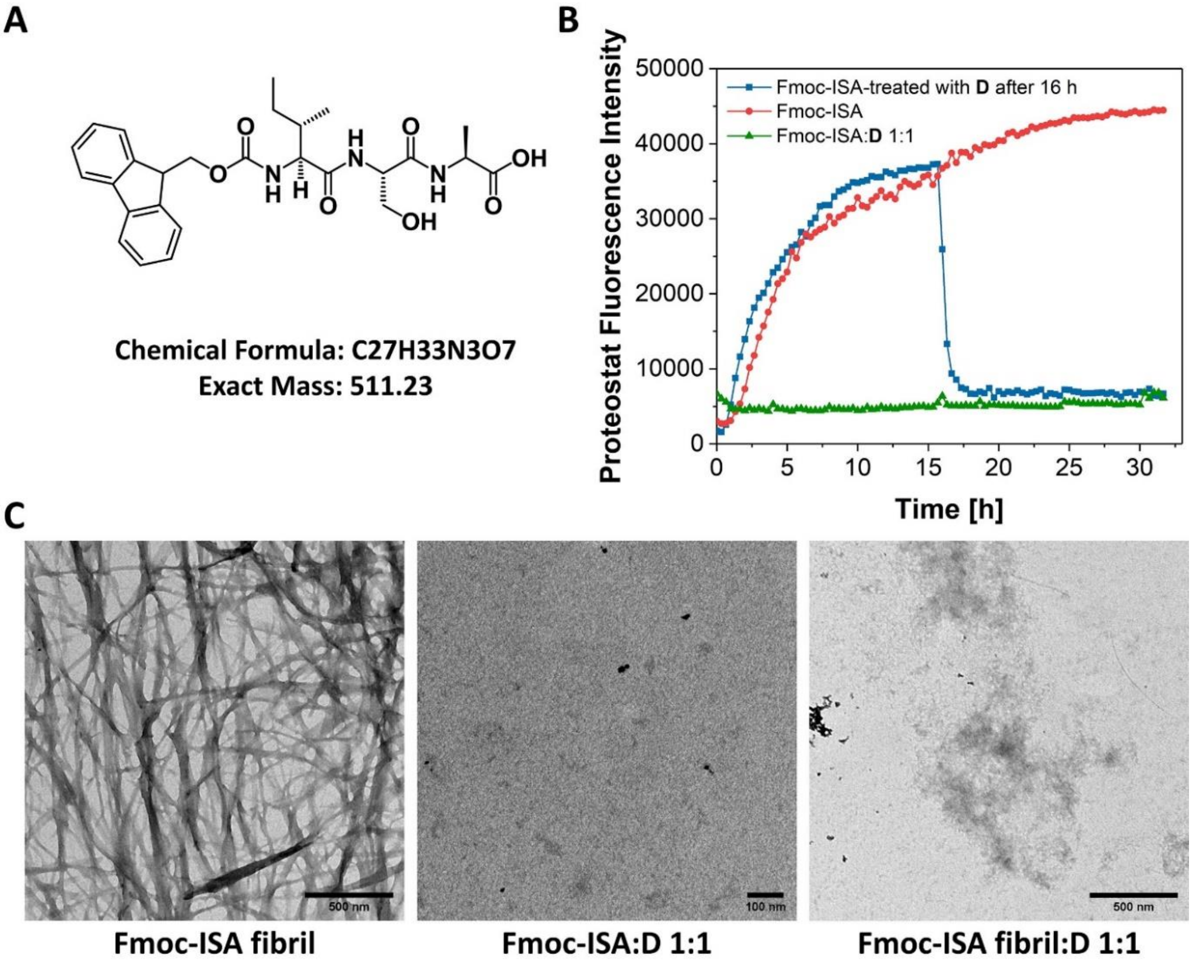

Figure S12. D inhibits the formation of the more hydrophobic amyloid-forming peptide Fmoc-ISA and disscociates already formed fibrils. (A) Chemical structure and molecular weight of Fmoc-ISA, (B) kinetics study of amyloid formation of Fmoc-ISA monomer, Fmoc-ISA monomer:**D** mixture (1:1) and Fmoc-ISA assembled firstly with subsequent addition of **D** (1:1 ratio) by using Proteostat protein aggregation assay (Fmoc-ISA 500 µM) and (C) TEM images revealing the formation of peptide nanofibers, inhibition of fibril formation and disassembly of already formed fibrils by equimolar ratio of B.

2.3 Defibrillation behavior of PAMAM (G3) on Aβ fibril

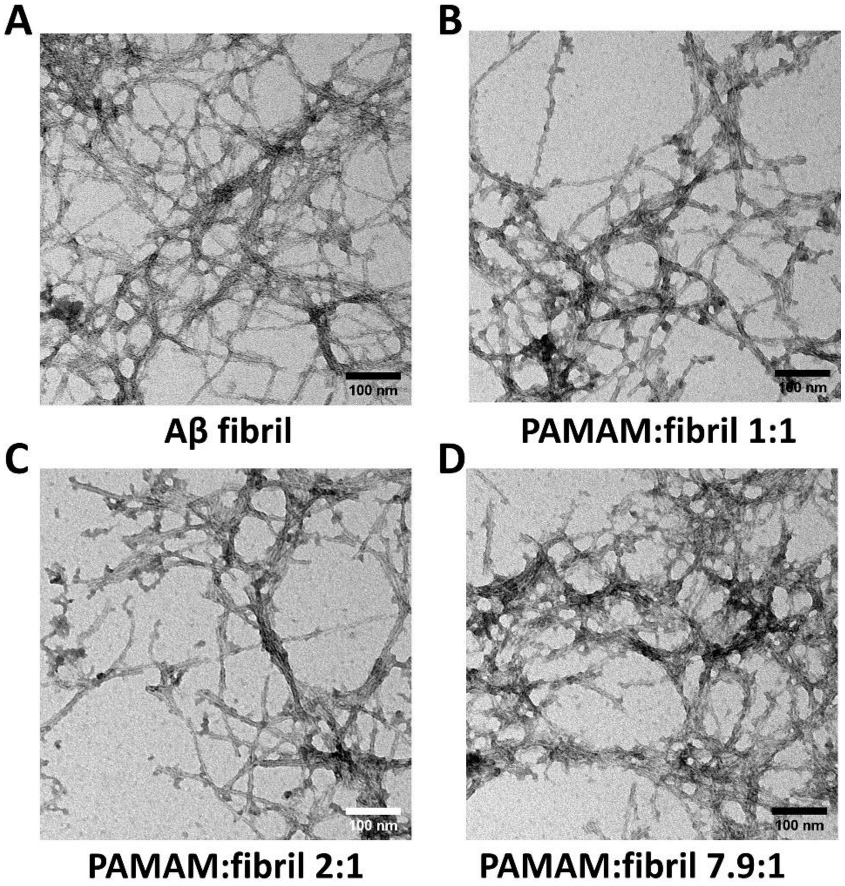

Figure S13. TEM images of Aβ fibrils, PAMAM dendrimer G3 with Aβ fibrils at different molar ratios (in all ratios, the Aβ fibril concentration is 20 µM). The scale bar is 100 nm.

3. Biological Characterization

3.1 *In vitro* cell uptake for primary neuronal cells

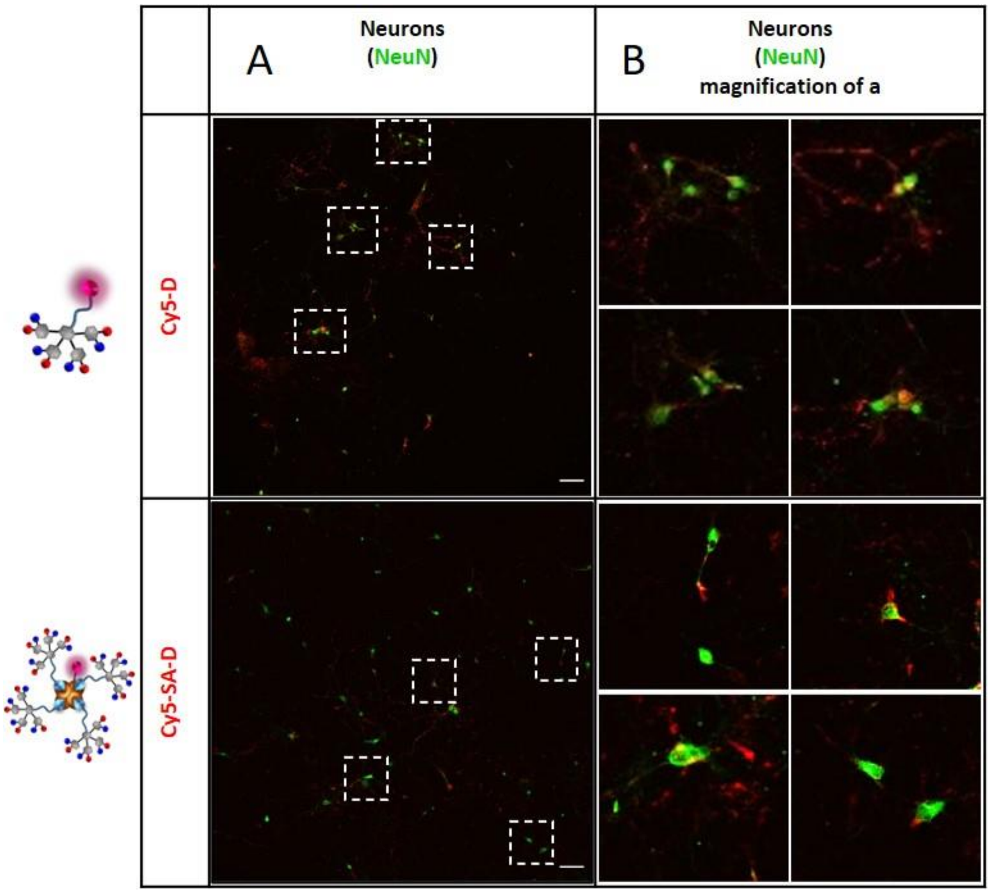

Figure S14. Cell uptake of Cy5-D and Cy5-SA-D by primary neurons. (A) Confocal imaging with low magnification of **Cy5-D** (red) and **Cy5-SA-D** (red) uptake in neurons *in vitro*. Cells were fixed, stained with neuron-specific antibody marker NeuN (green). (B) Magnification of **Cy5-D** and **Cy5-SA-D** positive neurons in boxes with dashed lines in (A). Scale bar is 50 µm.

3.2 *In vivo* Biodistribution

Mice were injected with **Cy5-D** (**3**) and **Cy5-SA-D** systemically via tail vein. 24 h after injection mice were perfused and organs were sliced and counterstained with nuclei dye DAPI. Imaging of the organs revealed a high uptake of dendron and **SA-D** in liver and spleen and low uptake in kidney, lung and heart.

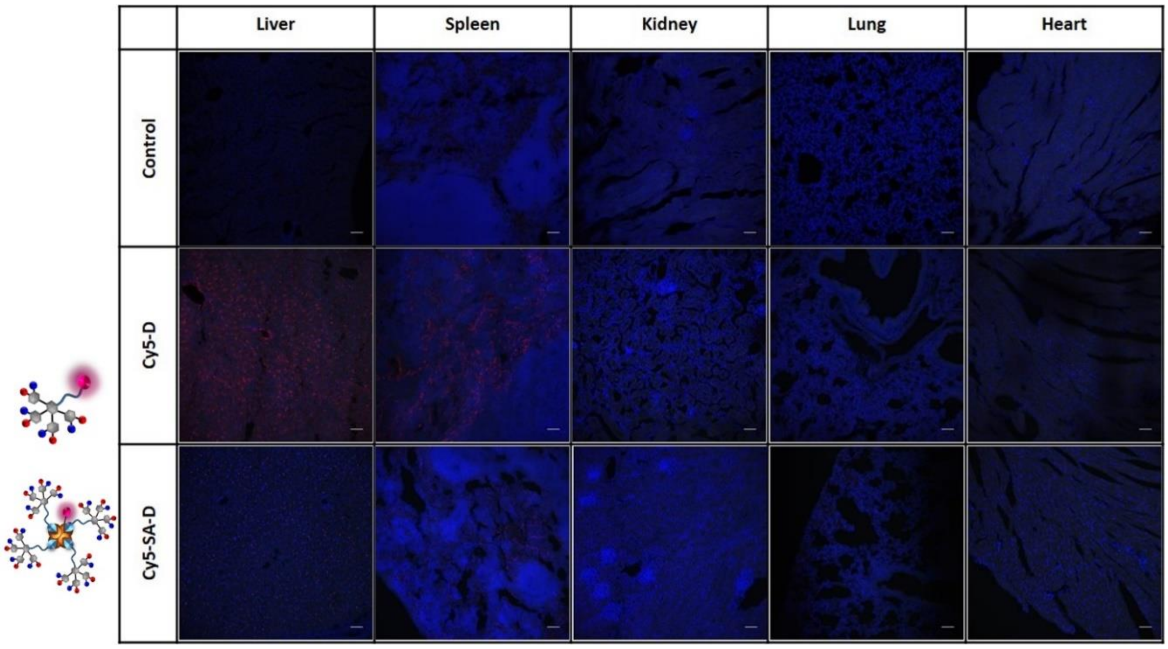

Figure S15. Biodistribution of **Cy5-D** and **Cy5-SA-D**. Mice were injected **Cy5-D** (red), **Cy5-SA-D** (red) or PBS for control. After 24 h mice were perfused and organs sliced, fixed and imaged. The scale is 50 µm.

3.3 Confidence Intervals for the data from Figure 5A, 7A and 7D

Figure 5A: Results: Brain endothelial cells (bEnd.3). 95% CI was 90.3 – 97.0% (**D** 20 µM), 75.9 – 97.3% (**D** 40 µM), 83.1 – 99.1% (**SA-D** 2.5 µM) and 90.0 – 103% (**SA-D** 5 µM).

Figure 7A: Results: 95% CI was 63.8 – 74.4% (Aβ 5 µM, 24 h), 24.5 – 65.3% (Aβ 5 µM, 48 h), 21.4 – 52.2% (Aβ 5 µM, 72 h), 89 – 102% (**D**:Aβ 1:1 5 µM, 24 h), 81.8 – 115% (**D**:Aβ 1:1 5 µM, 48 h), 85.6 – 97.8% (**D**:Aβ 1:1 5 µM, 72 h), 1.48 – 7.65% (PAMAM:Aβ 1:1 5 µM, 48 h), 0.45 – 1.08% (PAMAM:Aβ 1:1 5 µM, 72 h), 95% CI 9.59 -34.9 (Stauro, 24 h), 14.3 – 24.5 (Stauro, 48 h), 1.37 – 7.24% (Stauro, 72 h). Data are presented as mean with SEM. Dead cell control: cell toxin staurosporine (Stauro).

Figure 7D: Results: 95% CI 158 – 192% (NMDA 1mM), 114 – 130 (Aβ 5 µM), 97 – 106% (**D**:Aβ 1:1, Aβ 5 µM). 92.1 – 106% (**D** 5 µM) and 91.5 – 106% (control).

3.4 Primary neuronal cytotoxicity study of **D** and **SA-D** with Aβ

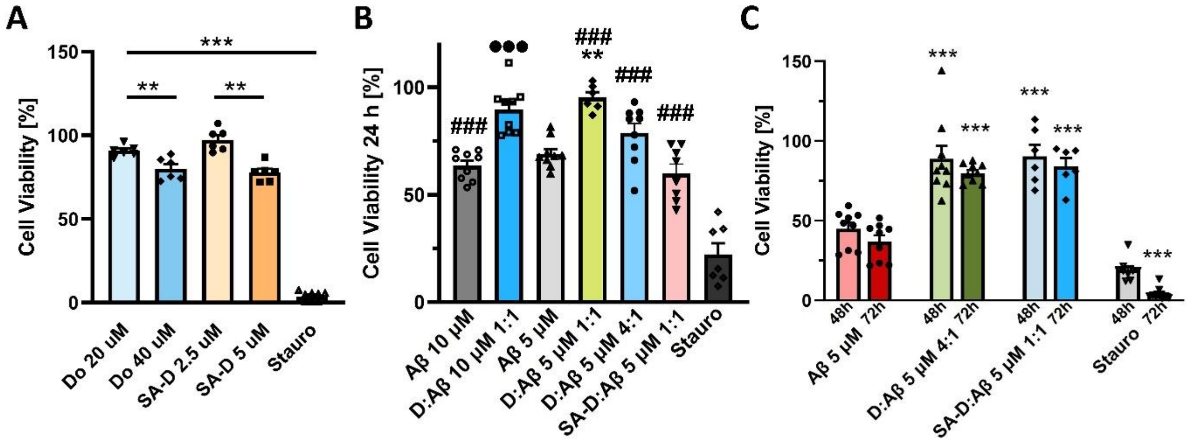

Figure S16. Cell viability of primary neuronal cells after (A) treatment with **D** and **SA-D**, (B) treatment with Aβ, **D**:Aβ, **SA-D**:Aβ complexes with different molar ratios and respective controls for 24 h and (c) treatment with either Aβ, **D**:Aβ 4:1, **SA-D**:Aβ 1:1 complexes for 48 and 72 h. Cell vitality was assessed by CellTiterGlo Assay and is presented in %. Data are presented as mean with SEM. n ≥ 6; one-way ANOVA. For panel A: ns > 0.05, * $p \leq 0.05$, ** $p \leq 0.01$, *** $p \leq 0.001$. For panel B: ** $p \leq 0.01$ versus Aβ 5 µM group, ••• $p \leq 0.001$ versus Aβ 10 µM group, ### $p \leq 0.001$ versus Stauro group. For panel c: *** $p \leq 0.001$ versus Aβ 5 µM 48 and 72 h groups. Results: (A) 95% CI was 87.5 – 94.4% (D 20 µM), 72.9 – 87.2% (**D** 40 µM), 90.3 – 104% (**SA-D** 2.5 µM) and 72.1 – 83.4% (**SA-D** 5 µm), (B) 95% CI was 58.5 – 68.9% (Aβ 10 µM), 95% CI 63.8 – 74.4 95% (Aβ 5 µM), 95% CI 9.59 -34.9 (Stauro), (C) 95% CI was 24.5 – 65.3% (Aβ 5 µM, 48 h), 21.4 – 52.2% (Aβ 5 µM, 72 h), 81.8 – 115% (**D**:Aβ 5 µM 4:1, 48 h), 75.4 – 84.4% (**D**:Aβ 5 µM 4:1, 72 h), 72 – 109% (**SA-D**:Aβ 5 µM 1:1, 48 h), 71.2 – 97.2% (**SA-D**:Aβ 5 µM 1:1, 72 h), 14.3 – 24.5% (Stauro, 48 h), 1.37 – 7.24% (Stauro, 72 h).

3.5 Cytotoxicity of PAMAM dendrimer (G3) on primary murine neuronal cells

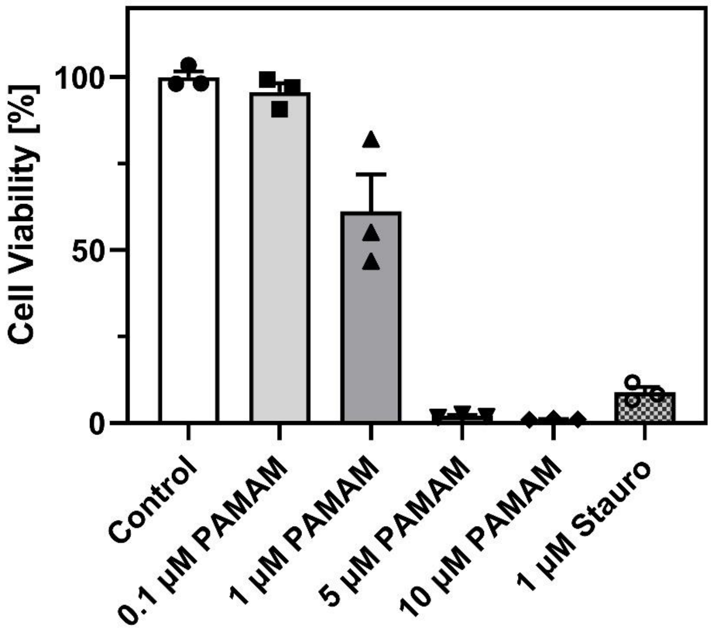

Figure S17. Different concentrations of PAMAM dendrimer (G3) were incubated with primary murine neuronal cells for 24 h. Cell vitality was assessed by CellTiterGlo Assay and is presented in %. Data are presented as mean with SEM. n = 3.

4. Movies S1-S6

Movie S1.

Control_GFAP/DAPI

Movie S2.

Cy5-D_GFAP/DAPI

Movie S3.

Cy5-SA-D_GFAP/DAPI

Movie S4.

Control_NeuN/DAPI

Movie S5.

Cy5-D_ NeuN/DAPI

Movie S6.

Cy5-SA-D_ NeuN/DAPI
